# Assessment of the Protocol-Guided Rapid Evaluation of Veterans Experiencing New Transient Neurological Symptoms (PREVENT) Program for Improving Quality of Care for Transient Ischemic Attack: A Nonrandomized Cluster Trial

**DOI:** 10.1001/jamanetworkopen.2020.15920

**Published:** 2020-09-08

**Authors:** Dawn M. Bravata, Laura J. Myers, Anthony J. Perkins, Ying Zhang, Edward J. Miech, Nicholas A. Rattray, Lauren S. Penney, Deborah Levine, Jason J. Sico, Eric M. Cheng, Teresa M. Damush

**Affiliations:** 1 Veterans Affairs Health Services Research and Development, Precision Monitoring to Transform Care Quality Enhancement Research Initiative, Department of Veterans Affairs, Indianapolis, Indiana; 2 Veterans Affairs Health Services Research and Development, Center for Health Information and Communication, Richard L. Roudebush VA Medical Center, Indianapolis, Indiana; 3 Department of Internal Medicine, Indiana University School of Medicine, Indianapolis; 4 Department of Neurology, Indiana University School of Medicine, Indianapolis; 5 Regenstrief Institute, Indianapolis, Indiana; 6 Department of Biostatistics, Indiana University School of Medicine, Indianapolis; 7 now with Department of Biostatistics, College of Public Health, University of Nebraska Medical Center, Omaha; 8 South Texas Veterans Health Care System, San Antonio; 9 Department of Medicine, University of Texas Health, San Antonio; 10 Department of Medicine, University of Michigan School of Medicine, Ann Arbor; 11 Clinical Epidemiology Research Center, VA Connecticut Healthcare System, West Haven; 12 VA Neurology Service, VA Connecticut Healthcare System, West Haven; 13 Department of Internal Medicine, Yale University School of Medicine, New Haven, Connecticut; 14 Department of Neurology and Center for Neuroepidemiology and Clinical Neurological Research, Yale University School of Medicine, New Haven, Connecticut; 15 Department of Neurology, VA Greater Los Angeles Healthcare System, Los Angeles, California; 16 Department of Neurology, David Geffen School of Medicine, University of California, Los Angeles

## Abstract

**Importance:**

Patients with transient ischemic attack (TIA) are at high risk of recurrent vascular events. Timely management can reduce that risk by 70%; however, gaps in TIA quality of care exist.

**Objective:**

To assess the performance of the Protocol-Guided Rapid Evaluation of Veterans Experiencing New Transient Neurological Symptoms (PREVENT) intervention to improve TIA quality of care.

**Design, Setting, and Participants:**

This nonrandomized cluster trial with matched controls evaluated a multicomponent intervention to improve TIA quality of care at 6 diverse medical centers in 6 geographically diverse states in the US and assessed change over time in quality of care among 36 matched control sites (6 control sites matched to each PREVENT site on TIA patient volume, facility complexity, and quality of care). The study period (defined as the data period) started on August 21, 2015, and extended to May 12, 2019, including 1-year baseline and active implementation periods for each site. The intervention targeted clinical teams caring for patients with TIA.

**Intervention:**

The quality improvement (QI) intervention included the following 5 components: clinical programs, data feedback, professional education, electronic health record tools, and QI support.

**Main Outcomes and Measures:**

The primary outcome was the without-fail rate, which was calculated as the proportion of veterans with TIA at a specific facility who received all 7 guideline-recommended processes of care for which they were eligible (ie, anticoagulation for atrial fibrillation, antithrombotic use, brain imaging, carotid artery imaging, high- or moderate-potency statin therapy, hypertension control, and neurological consultation). Generalized mixed-effects models with multilevel hierarchical random effects were constructed to evaluate the intervention associations with the change in the mean without-fail rate from the 1-year baseline period to the 1-year intervention period.

**Results:**

Six facilities implemented the PREVENT QI intervention, and 36 facilities were identified as matched control sites. The mean (SD) age of patients at baseline was 69.85 (11.19) years at PREVENT sites and 71.66 (11.29) years at matched control sites. Most patients were male (95.1% [154 of 162] at PREVENT sites and 94.6% [920 of 973] at matched control sites at baseline). Among the PREVENT sites, the mean without-fail rate improved substantially from 36.7% (58 of 158 patients) at baseline to 54.0% (95 of 176 patients) during a 1-year implementation period (adjusted odds ratio, 2.10; 95% CI, 1.27-3.48; *P* = .004). Comparing the change in quality at the PREVENT sites with the matched control sites, the improvement in the mean without-fail rate was greater at the PREVENT sites than at the matched control sites (36.7% [58 of 158 patients] to 54.0% [95 of 176 patients] [17.3% absolute improvement] vs 38.6% [345 of 893 patients] to 41.8% [363 of 869 patients] [3.2% absolute improvement], respectively; absolute difference, 14%; *P* = .008).

**Conclusions and Relevance:**

The implementation of this multifaceted program was associated with improved TIA quality of care across the participating sites. The PREVENT QI program is an example of a health care system using QI strategies to improve performance, and may serve as a model for other health systems seeking to provide better care.

**Trial Registration:**

ClinicalTrials.gov Identifier: NCT02769338

## Key Points

**Question:**

Is the Protocol-Guided Rapid Evaluation of Veterans Experiencing New Transient Neurological Symptoms (PREVENT) program associated with improved care for transient ischemic attack?

**Findings:**

This nonrandomized cluster trial with matched controls (including 6 PREVENT sites and 36 matched control sites) evaluated quality of care for transient ischemic attach across 7 guideline-recommended processes of care measured by the without-fail rate. Over the course of a 1-year implementation period, the mean without-fail rate improved 17% at the 6 PREVENT sites and only 3% at 36 matched control sites.

**Meaning:**

These findings suggest that implementation of a multicomponent program such as PREVENT may improve quality of care for transient ischemic attack.

## Teaser

This nonrandomized cluster trial with matched controls implements the Protocol-Guided Rapid Evaluation of Veterans Experiencing New Transient Neurological Symptoms (PREVENT) intervention to improve transient ischemic attack quality of care.

## Introduction

Approximately 8500 veterans with transient ischemic attack (TIA) or ischemic stroke are cared for in Department of Veterans Affairs (VA) emergency departments (EDs) or inpatient wards annually in the United States.^1^ Patients with TIA generally present with transient neurological symptoms of a presumed ischemic cause.^2^ Patients with TIA are at a high risk of recurrent vascular events^3,4,5^; however, delivery of timely TIA care can reduce that risk by up to 70%.^6,7,8,9^ Despite the known benefits of timely TIA care, gaps in TIA quality of care exist in both private-sector US hospitals^10^ and VA facilities.^11,12^

In a learning health care system, “clinical informatics, incentives, and culture are aligned to promote continuous improvement and innovation, with best practices seamlessly embedded in the delivery process and new knowledge captured as an integral by-product of the delivery experience.”^13^^(p136)^ Within a learning health care system, health care teams respond to quality problems by using quality improvement (QI) strategies and systems redesign approaches to improve performance, depending on the complexity and scope of the problem.^14^ The objective of the Protocol-Guided Rapid Evaluation of Veterans Experiencing New Transient Neurological Symptoms (PREVENT) trial was to evaluate a multicomponent QI intervention to improve the quality of TIA care.^15^ The PREVENT intervention was designed to align with the learning health care system model.^13,15^

## Methods

**Figure 1.**
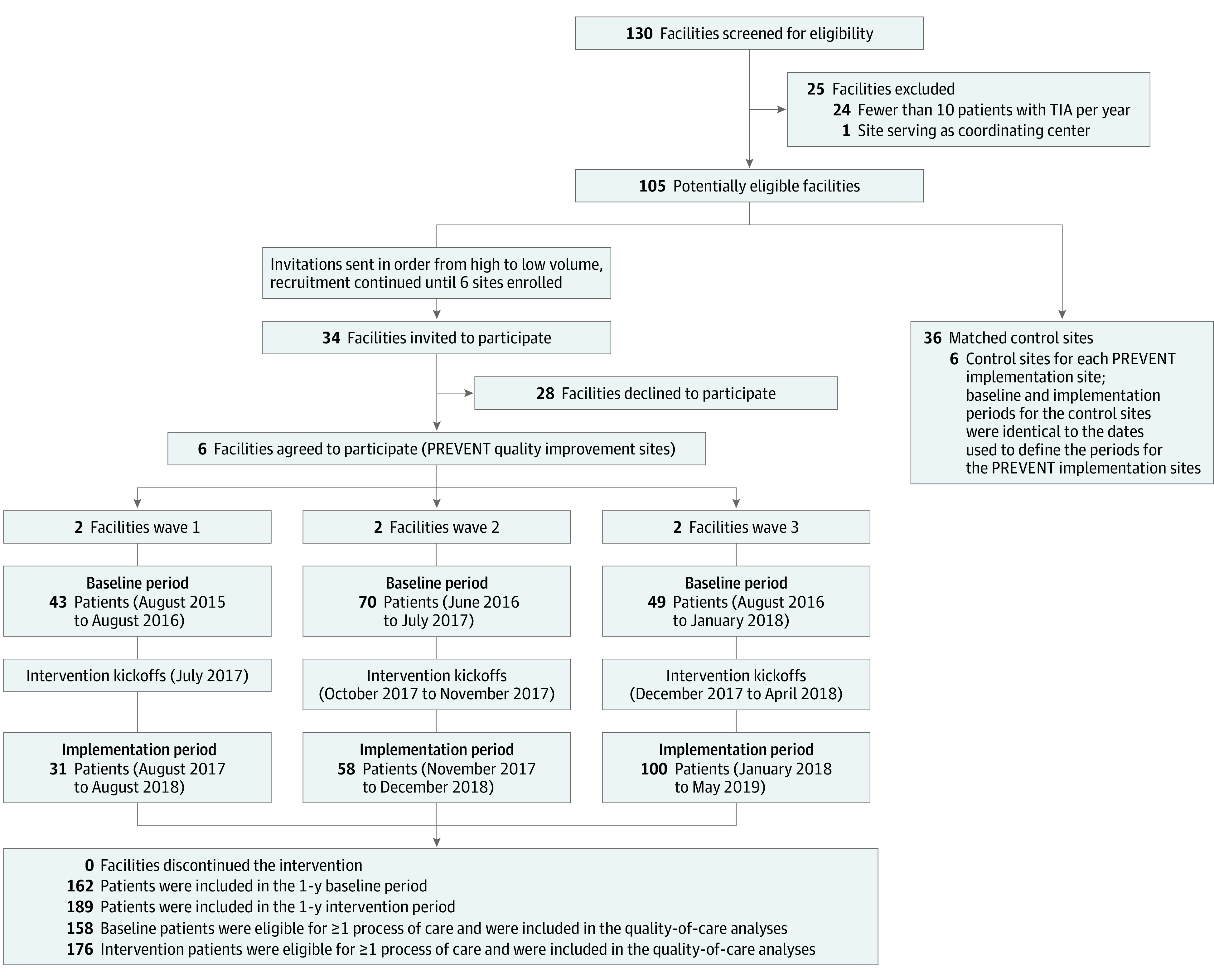
Study Flow Diagram Shown is the nonrandomized cluster trial with matched controls study design, with 2 facilities per wave and 6 matched control sites for every PREVENT (Protocol-Guided Rapid Evaluation of Veterans Experiencing New Transient Neurological Symptoms) site. TIA indicates transient ischemic attack.

This nonrandomized cluster trial with matched controls^16,17^ included 6 participating sites (referred to as PREVENT sites), where active implementation was initiated in 3 waves, with 2 facilities per wave (Figure 1), and 36 matched control sites (Trial protocol in Supplement 1). The cluster design permitted the dissemination of intervention resources to all intervention teams over time seeking to fundamentally improve the standard of care for all patients with TIA at participating sites.^16,17^ The intervention was at the facility level, but the unit of analysis was at the patient level. This study followed the Standards for Reporting Implementation Studies (StaRI) Statement. The study received human participant approval from the Indiana University School of Medicine Institutional Review Board and the Richard L. Roudebush VA Medical Center Research and Development Committee. A waiver of written informed consent was granted by the institutional review board for the collection of medical record data because the data were obtained retrospectively from administrative sources.

### Setting and Study Periods

Within the VA, quality measurement and systems redesign are integrated into the health care system within administrative and clinical operations.^18,19^ Although stroke care quality metrics are reported herein, there is no VA systemwide focus on TIA quality of care.

The site recruitment methods have been described.^15^ The VA hospitals were rank ordered in terms of quality of TIA care^12^; invitations to participate were sent beginning with facilities with the lowest performance and the highest TIA annual patient volume. Recruitment continued until 6 facilities agreed to participate. These 6 sites were in 6 geographically diverse states in the US. The PREVENT sites were pragmatically allocated to waves based on the ability to schedule baseline meetings.

Six control sites were matched to each of the 6 PREVENT sites based on TIA patient volume, facility complexity (ie, teaching status, intensive care unit level), and quality of care (measured by the without-fail rate, described below). The total number of matched control sites was 36.

The baseline period extended for 12 months before the baseline site visit at the participating facilities (Figure 1). The active implementation period began 1 month after the kickoff (Figure 1), which was the day that the site launched the program, providing 1 month for facility teams to initiate QI activities before assessment of outcomes began. The kickoffs were scheduled in waves, with 2 sites per wave; the first kickoff was on July 11, 2017, and the last one was on April 13, 2018. Therefore, the baseline and implementation periods were temporally staggered across the waves. The time between the baseline visit and the kickoff varied, depending on the availability of site team members to schedule the full-day kickoff. The period between the baseline visit and the kickoff was excluded from the analyses. The first baseline period (wave 1, site 1) began on August 21, 2015, and the last baseline period (wave 3, site 2) ended on January 30, 2018. The first implementation period began on August 11, 2017, and the final implementation period ended on May 12, 2019. The definition of the baseline period (specific start and end dates of the 1-year baseline period) for each matched control site was identical to the definition used for the PREVENT site to which the controls were matched.

### QI Intervention

The rationale and methods used to develop the PREVENT intervention have been described.^15^ The PREVENT intervention was designed on the basis of an assessment of TIA care performance at VA facilities nationwide, as well as an evaluation of barriers to and facilitators of TIA care performance using the following 4 information sources: baseline quality-of-care data,^12^ staff interviews,^20^ existing literature,^21,22,23,24^ and validated electronic quality measures.^12,25^

The PREVENT QI intervention targeted facility staff rather than patients. External facilitation was provided by the study team (D.M.B., L.J.M., E.J.M., N.A.R., and T.M.D.), primarily by a nurse with quality management expertise and secondarily by a physician (D.M.B.) with both cerebrovascular disease content knowledge and systems redesign experience. The rest of the study team members had complementary experience in implementation science, data science, and systems redesign. The composition of the participating facility teams varied across sites but generally included neurologists, emergency medicine practitioners, nurses, pharmacists, and radiologists; some teams also included hospitalists, clinicians, education staff, telehealth staff, and systems redesign staff.

The PREVENT intervention targeted clinical teams (not patients) and consisted of the following 5 components: clinical programs (including clinical pathways with educational materials and documentation tools), data feedback, professional education, electronic health record tools, and QI support (including a virtual collaborative).^15^ Active implementation of the PREVENT intervention involved a full-day kickoff during which the facility team explored their site-specific quality of care data to identify processes of care with the largest gaps in quality for the greatest number of patients. Using systems redesign approaches, facility team members brainstormed about barriers to providing the highest quality of care, identified solutions to address barriers, ranked solutions in terms of potential benefits and effort, and developed a site-specific action plan. Local QI plans were entered into the PREVENT web-based hub, and quality metrics were tracked, allowing teams to monitor performance over time. During the 1-year active implementation period, site teams joined monthly virtual collaborative conferences, a forum for cross-site conversation and discussion, including sharing progress on action plans, articulating goals for the next month, and reviewing any new evidence or tools. The design of the PREVENT QI program advanced the following 3 aspects of a learning health care system: learning from data (via the PREVENT hub, which unlike static performance dashboards allowed teams to examine and interact with their performance data to explore hypotheses, plan QI activities, and evaluate change over time), learning from each other (via the virtual collaborative conferences), and sharing best practices (via the growing library of diverse resources on the PREVENT hub).^15^

### Outcomes

The primary outcome was the without-fail rate (sometimes referred to as defect-free care^26,27^), which is an all-or-none measure of quality of care.^28^ The without-fail rate is calculated as the proportion of veterans with TIA at a specific facility who received all of the processes of care for which they were eligible from among the following 7 guideline-recommended processes of care: anticoagulation for atrial fibrillation within 7 days of discharge, antithrombotic use within 2 days after the event, brain imaging within 2 days after the event or 1 day before the index TIA, carotid artery imaging within 2 days after the event or 6 months before the event, high- or moderate-potency statin therapy within 7 days of discharge, hypertension control over the 90 days after discharge, and neurological consultation by day 1 after the TIA.^15,25^ The processes of care were based on electronic health record data using validated algorithms.^12,25^ The without-fail rate was based on guideline-recommended processes of care^6,29^ and has been associated with improved outcomes.^30^ Given the all-or-none nature of the without-fail rate, it can be a difficult outcome to change, and even small improvements in the absolute rate may reflect substantial changes in practice at the facility level.^28^

The prespecified secondary outcomes were the consolidated measure of quality (number of passes divided by number of opportunities) and the individual processes of care that were included in both the without-fail rate and the consolidated measure of quality. eTable 1 in Supplement 2 lists the numerator and denominator definitions for each process of care.

We also examined the 90-day recurrent stroke rate, 90-day recurrent vascular event rate, and 90-day mortality rate (eTable 2 in Supplement 2 lists the diagnosis codes).^7,8,24^ Recurrent stroke (defined as an ischemic stroke in the emergency department [ED] or resulting in an inpatient admission within 90 days of discharge of the index TIA event) and recurrent vascular events^31^ (defined as an ED visit or inpatient admission within 90 days of discharge for congestive heart failure, myocardial infarction or acute coronary syndrome, ischemic stroke, TIA, ventricular arrhythmia, or death) were identified via primary diagnosis codes in the ED or inpatient setting using a combination of both VA and fee-basis data (which describe health care in non–VA facilities that was paid for by the VA). Therefore, recurrent stroke or recurrent vascular events that occurred in community hospitals but were not paid for by the VA were not included. The 90-day all-cause mortality rate (defined as death from any cause within 90 days of presentation for the index event) was obtained from the VA Vital Status File.^32^

### Identification of Patients With TIA for Calculation of the Quality Measures

Details of our methods have been described.^15^ We retrospectively identified veterans with TIA who were cared for in the ED or inpatient setting based on primary discharge codes for TIA (*International Classification of Diseases, Ninth Revision* [*ICD-9*] 435.x, excluding 435.2; and *International Statistical Classification of Diseases and Related Health Problems, Tenth Revision *[*ICD-10*] codes G45.0, G45.1, G45.8, G45.9, and I67.848).^25^ Patients with possible TIA events who were cared for entirely in the outpatient setting (without either an ED visit or inpatient admission) were not included. Comparing this electronic health record approach with chart review, 95.0% (436 of 459) of the patients classified as having a TIA using the electronic health record approach were found to have a TIA diagnosis in the chart review, 4.1% (19 of 459) of cases had a chart review diagnosis of ischemic stroke, and 0.9% (4 of 459) of patients had another diagnosis (neither stroke nor TIA).^25^ Patients cared for in hospital observation settings were classified as inpatients. Patients cared for in an ED without an inpatient or observation unit admission were classified as ED-only patients.

### Data Sources

Process of care data were obtained from the VA Corporate Data Warehouse (CDW), which includes a broad range of information from the VA electronic medical record system, known as Veterans Information Systems and Technology Architecture (VistA). VistA is the electronic medical record system that is used across the entire VA system nationwide and includes clinical and administrative functionality.^33^ The CDW data included inpatient and outpatient data files (eg, clinical encounters with associated diagnostic and procedure codes) in the 5 years before the event to identify medical history,^34^ health care use, and receipt of procedures (*Current Procedural Terminology*, Healthcare Common Procedures Coding System, and *ICD-9* and *ICD-10* procedure codes). The CDW data were also used for vital signs, laboratory data, allergies, imaging, orders, medications, and clinical consultations. Most CDW data are updated nightly. Fee-basis data were also used to identify inpatient and outpatient health care use and medical history.

### Statistical Analysis

The Fisher exact test was used to compare whether categorical variables differed between the PREVENT sites and the matched control sites as well as between baseline and implementation periods. Two-sample *t* tests or Wilcoxon rank sum tests were used to ascertain whether continuous outcomes differed between the PREVENT sites and the matched control sites as well as between baseline and implementation periods. Generalized mixed-effects models at the patient level, with random effects for site and fixed effects for wave, were used to analyze the PREVENT intervention associations.^35^ Separate risk-adjustment models were constructed for each process of care, for the without-fail rate, and for the consolidated measure of quality. Fully risk-adjusted models included site, wave, and the specific patient characteristics that were associated with the particular outcome of interest (eg, the without-fail rate). Variables used in the risk-adjusted models are listed in eTable 3 in Supplement 2. All analyses were performed using SAS Enterprise Guide, version 7.11 (SAS Institute Inc).

The primary analysis compared the mean without-fail rate in the 1-year baseline period vs the 1-year active implementation period. We also compared the change in the without-fail rate from baseline to 1 year between the PREVENT sites and the matched control sites to ameliorate the potential association of temporal trends in care that may have confounded the assessment of the intervention association within the cluster design.

The secondary analyses included a comparison of baseline vs active implementation for the consolidated measure of quality, the 7 guideline-recommended processes of care, and 90-day outcomes. The primary analyses were focused on the first TIA event per patient during the study period (eg, a patient with a TIA event in both baseline and active implementation was only included in the baseline period). We also conducted sensitivity analyses in which we included all TIA events (ie, not restricted to the first TIA event) and excluded patients 90 years or older (because care for such patients may appropriately not include all of the processes of care that are included in the without-fail rate). The testing was 2 sided, with the threshold of statistical significance set at *P* < .05.

### Power and Sample Size

The methods used for the sample size design and power calculation for this nonrandomized cluster trial with matched controls have been reported.^35^ Briefly, the 6-site study was designed to provide greater than 90% power to detect an improvement in the mean facility without-fail rate from 25% during the baseline period to greater than 45% during the active implementation period. The goal for the sample size was to recruit sites with at least 50 patients with TIA per year; however, based on the original sample size calculations, power would be preserved with at least 30 patients with TIA per year for an effect size of 20% increase in the mean facility without-fail rate. This study was not powered to detect differences in patient outcomes (eg, mortality, recurrent events).

## Results

The mean (SD) age of participants at baseline was 69.9 (11.2) years at PREVENT sites and 71.6 (11.3) years at matched control sites. Most patients were male (95.1% [154 of 162] at the PREVENT sites and 94.6% [920 of 973] at the matched control sites at baseline).

The 6 PREVENT sites were geographically diverse, including sites in the West, Northeast, Southeast, and Midwest. The annual TIA patient volume varied across sites, ranging from 13 to 43 patients. The sites varied in terms of the infrastructure to provide care for patients with TIA and to improve quality of care. Five of the 6 teams were led by a neurologist (1 team was co-led by a facility-based systems redesign specialist), and 1 team was led by an emergency medicine quality management nurse. All sites had neurology attending staff coverage, but the neurology service varied across sites in terms of size (the median neurology full-time equivalent [FTE] was 4.7; interquartile range, 3.1-6.7), admitting service status (vs consulting service only), and teaching status (neurology resident training program). All sites had an ED (the median ED FTE was 7.1; interquartile range, 6.1-8.9). All sites had pharmacists as team members. Although none of the sites had preexisting teams or projects monitoring or improving TIA care, all teams had at least 1 member with previous QI experience. The PREVENT sites and the matched control sites were similar in terms of neurology and ED FTEs (eTable 4 in Supplement 2).

Table 1 lists the patient characteristics of the 6 PREVENT sites (during baseline and active implementation periods) and the matched control sites, including CHA_2_DVAS_2_c (congestive heart failure or left ventricular dysfunction, hypertension, age 75 years or older [doubled], diabetes, stroke [doubled]–vascular disease, age 65 to 74 years, and sex category [female]),^36^ HAS-BLED (hypertension, abnormal renal/liver function, stroke, bleeding history or predisposition, labile international normalized ratio [if taking warfarin sodium], elderly [eg, age >65 years, frail condition], drugs [eg, aspirin, nonsteroidal anti-inflammatory drugs]/alcohol concomitantly),^37^ and APACHE (Acute Physiology and Chronic Health Evaluation)^38^ values. Most patients with TIA were admitted to the hospital, but the hospital admission rate was higher in the PREVENT sites compared with the matched control sites (77.2% [125 of 162 patients] vs 64.5% [628 of 973 patients], *P* = .002). Comorbidities were common, including hypertension in 79.6% (129 of 162) of patients, hyperlipidemia in 67.9% (110 of 162), and diabetes in 48.1% (78 of 162) (Table 1). The patient characteristics were similar between patients cared for in the PREVENT sites and the matched control sites (Table 1). Within the PREVENT sites, the characteristics of patients were similar during the baseline and active implementation periods (Table 1).

**Table 1.** Baseline Patient Characteristics

Baseline patient characteristic	No. (%)				*P* value	
	Matched control sites		PREVENT sites		Matched control sites vs PREVENT sites	PREVENT sites baseline vs implementation
	Baseline (n = 973)	Implementation (n = 968)	Baseline (n = 162)	Implementation (n = 189)		
**Index event**						
Admitted vs ED for index event						
ED only	345 (35.5)	310 (32.0)	37 (22.8)	36 (19.0)	.002	.43
Admitted to the hospital	628 (64.5)	658 (68.0)	125 (77.2)	153 (81.0)		
Weekday presentation	782 (80.4)	759 (78.4)	130 (80.2)	143 (75.7)	>.99	.37
Left against medical advice	38 (3.9)	52 (5.4)	4 (2.5)	9 (4.8)	.50	.40
**Demographic characteristics**						
Age, y						
Mean (SD)	71.7 (11.3)	71.4 (10.9)	69.9 (11.2)	68.8 (11.3)	.06	.38
Median (IQR)	71.0 (65.0-80.0)	71.0 (65.0-78.0)	69.0 (63.0-76.0)	69.0 (61.0-75.0)	.02	.52
Male sex	920 (94.6)	914 (94.4)	154 (95.1)	181 (95.8)	>.99	.80
Race						
White	770 (79.1)	727 (75.1)	116 (71.6)	124 (65.6)	.11	.65
Black	145 (14.9)	184 (19.0)	37 (22.8)	50 (26.5)		
Asian	5 (0.5)	9 (0.9)	0	2 (1.1)		
Other	4 (0.4)	8 (0.8)	1 (0.6)	1 (0.5)		
Unknown	49 (5.0)	40 (4.1)	8 (4.9)	12 (6.3)		
Hispanic ethnicity	45 (4.6)	58 (6.0)	18 (11.1)	16 (8.5)	.002	.47
**Medical history**						
TIA outpatient encounter in prior 30 d^a^	56 (5.8)	38 (3.9)	7 (4.3)	11 (5.8)	.58	.63
Stroke in prior 30 d	65 (6.7)	57 (5.9)	7 (4.3)	10 (5.3)	.30	.81
Diabetes	404 (41.5)	411 (42.5)	78 (48.1)	78 (41.3)	.12	.20
Atrial fibrillation	171 (17.6)	178 (18.4)	35 (21.6)	32 (16.9)	.23	.28
Myocardial infarction	71 (7.3)	79 (8.2)	14 (8.6)	11 (5.8)	.52	.41
Congestive heart failure	165 (17.0)	164 (16.9)	22 (13.6)	21 (11.1)	.36	.52
Carotid endarterectomy or stent	11 (1.1)	6 (0.6)	1 (0.6)	0	>.99	.46
Chronic obstructive pulmonary disease	207 (21.3)	202 (20.9)	27 (16.7)	36 (19.0)	.21	.58
Peripheral arterial disease	163 (16.8)	153 (15.8)	20 (12.3)	29 (15.3)	.17	.44
Dementia	81 (8.3)	63 (6.5)	16 (9.9)	12 (6.3)	.54	.24
Chronic kidney disease	160 (16.4)	175 (18.1)	29 (17.9)	27 (14.3)	.65	.38
Dialysis	13 (1.3)	21 (2.2)	6 (3.7)	1 (0.5)	.04	.05
Cancer	123 (12.6)	100 (10.3)	15 (9.3)	21 (11.1)	.25	.60
Hypertension	716 (73.6)	746 (77.1)	129 (79.6)	152 (80.4)	.12	.89
Hyperlipidemia	584 (60.0)	610 (63.0)	110 (67.9)	120 (63.5)	.07	.43
Speech deficit	44 (4.5)	57 (5.9)	13 (8.0)	12 (6.3)	.08	.68
Motor deficit, hemiplegia	128 (13.2)	163 (16.8)	25 (15.4)	40 (21.2)	.46	.21
Sleep apnea	177 (18.2)	237 (24.5)	34 (21.0)	44 (23.3)	.39	.70
Alcohol dependence	72 (7.4)	77 (8.0)	14 (8.6)	21 (11.1)	.63	.48
Depression	209 (21.5)	229 (23.7)	45 (27.8)	42 (22.2)	.08	.27
History of venous thromboembolism	30 (3.1)	38 (3.9)	12 (7.4)	7 (3.7)	.01	.16
Intracranial hemorrhage	57 (5.9)	52 (5.4)	13 (8.0)	10 (5.3)	.29	.39
Gastrointestinal bleeding	7 (0.7)	5 (0.5)	2 (1.2)	1 (0.5)	.62	.60
Migraine	25 (2.6)	28 (2.9)	8 (4.9)	9 (4.8)	.12	>.99
Medications before index event						
Antihypertensives	818 (84.1)	810 (83.7)	142 (87.7)	160 (84.7)	.29	.44
Statin	785 (80.7)	808 (83.5)	135 (83.3)	169 (89.4)	.45	.12
Aspirin	733 (75.3)	687 (71.0)	132 (81.5)	149 (78.8)	.09	.59
Warfarin sodium	125 (12.8)	86 (8.9)	11 (6.8)	17 (9.0)	.03	.55
CHA_2_DVAS_2_c, mean (SD)^b^	3.25 (1.47)	3.26 (1.49)	3.31 (1.39)	3.11 (1.33)	.62	.15
HAS-BLED, mean (SD)^c^	2.23 (1.03)	2.24 (1.08)	2.20 (1.09)	2.06 (1.02)	.77	.22
Charlson Comorbidity Index score						
Mean (SD)	2.9 (2.7)	2.9 (2.7)	2.8 (2.8)	2.7 (2.8)	.75	.59
Median (IQR)	2.0 (1.0-4.0)	3.0 (2.0-4.0)	2.0 (0.0-4.0)	2.0 (0.0-4.0)	.51	.60
Smoker	265 (27.2)	259 (26.8)	44 (27.2)	61 (32.3)	>.99	.35
Palliative care, hospice	44 (4.5)	31 (3.2)	3 (1.9)	3 (1.6)	.14	>.99
**Present on admission**						
Concomitant myocardial infarction	23 (2.4)	24 (2.5)	8 (4.9)	5 (2.6)	.07	.27
Concomitant congestive heart failure	15 (1.5)	19 (2.0)	4 (2.5)	1 (0.5)	.33	.19
**Laboratory and vital signs**						
APACHE^d^						
Mean (SD)	9.5 (6.7)	10.2 (6.9)	10.2 (7.4)	9.2 (6.1)	.25	.17
Median (IQR)	9.0 (4.0-14.0)	9.0 (5.0-14.0)	9.0 (5.0-14.0)	9.0 (4.0-12.0)	.53	.57
First systolic blood pressure, mm Hg						
Mean (SD)	147.7 (25.8)	148.5 (25.8)	145.3 (24.1)	142.8 (25.2)	.27	.34
Median (IQR)	147.0 (129.0-165.0)	148.0 (130.0-164.0)	144.0 (128.0-162.0)	142.0 (125.0-159.0)	.24	.39
First diastolic blood pressure, mm Hg						
Mean (SD)	80.3 (13.1)	81.2 (14.4)	81.0 (14.6)	80.2 (14.4)	.53	.62
Median (IQR)	80.0 (71.0-89.0)	81.0 (71.0-90.0)	80.0 (72.0-90.0)	79.0 (71.0-90.0)	.85	.79
Average systolic blood pressure 90 d after discharge, mm Hg						
Mean (SD)	131.4 (16.4)	131.2 (15.8)	130.3 (15.6)	127.5 (14.1)	.49	.10
Median (IQR)	130.4 (120.0-141.0)	130.0 (121.3-139.5)	130.0 (120.0-138.9)	126.6 (118.0-136.3)	.57	.09
Average diastolic blood pressure 90 d after discharge, mm Hg						
Mean (SD)	74.5 (9.3)	73.7 (9.8)	74.6 (9.3)	73.1 (9.5)	.93	.17
Median (IQR)	74.5 (68.7-80.0)	74.0 (67.0-80.0)	75.0 (69.0-80.0)	73.0 (67.0-80.0)	.85	.21
**Health care use**						
Any inpatient admission in year before index event	303 (31.1)	280 (28.9)	55 (34.0)	43 (22.8)	.47	.02
Any ED visit in year before index event	598 (61.5)	587 (60.6)	89 (54.9)	104 (55.0)	.12	>.99
Primary care visit in 90 d after discharge	775 (79.7)	793 (81.9)	140 (86.4)	155 (82.0)	.05	.31
Neurology visit in 90 d after discharge	379 (39.0)	411 (42.5)	73 (45.1)	82 (43.4)	.14	.83

Abbreviations: APACHE, Acute Physiology and Chronic Health Evaluation; CHA_2_DVAS_2_c, congestive heart failure or left ventricular dysfunction, hypertension, age 75 years or older (doubled), diabetes, stroke (doubled)–vascular disease, age 65 to 74 years, and sex category (female); ED, emergency department; HAS-BLED, hypertension, abnormal renal/liver function, stroke, bleeding history or predisposition, labile international normalized ratio (if taking warfarin sodium), elderly (eg, age >65 years, frail condition), drugs (eg, aspirin, nonsteroidal anti-inflammatory drugs)/alcohol concomitantly; IQR, interquartile range; TIA, transient ischemic attack.

^a^ Patients were included in this cohort if they had a TIA event (defined as an ED visit or inpatient admission for TIA); outpatient visits were not used to identify the index TIA event. The first TIA ED visit or inpatient admission during the study period was classified as the index event. Some of the patients in the cohort had an outpatient encounter (eg, primary care visit) in the 30 days before the index TIA.

^b^ CHA_2_DVAS_2_c is a score that describes the risk of thromboembolism among patients with atrial fibrillation. Higher scores indicate greater risk of thromboembolism.^36^

^c^ HAS-BLED refers to a score that describes the risk of bleeding among patients who are anticoagulated. Higher scores indicate greater risk of bleeding.^37^

^d^ APACHE scoring system is a measure of physiological illness and has been shown to predict mortality risk among acutely ill patients. Higher scores indicate greater physiological illness.^38^

### Change in the Primary and Secondary Outcomes

**Figure 2.**
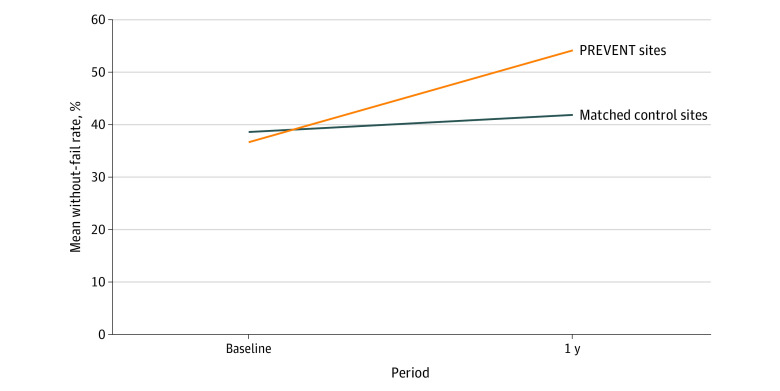
Change in TIA Quality of Care for the PREVENT Sites vs the Matched Control Sites The primary outcome was the without-fail rate, which is calculated as the proportion of veterans with transient ischemic attack (TIA) at a specific facility who received all 7 guideline-recommended processes of care for which they were eligible (ie, anticoagulation for atrial fibrillation, antithrombotic use, brain imaging, carotid artery imaging, high- or moderate-potency statin therapy, hypertension control, and neurological consultation). PREVENT indicates Protocol-Guided Rapid Evaluation of Veterans Experiencing New Transient Neurological Symptoms.

Among the PREVENT sites, the mean without-fail rate improved from 36.7% (58 of 158 patients) at baseline to 54.0% (95 of 176 patients) during the implementation period (odds ratio [OR] adjusted for facility and wave, 2.15; 95% CI, 1.34-3.45; *P* = .002) (Table 2); eTable 5 in Supplement 2 lists changes in individual facility performance). The improvement in the mean without-fail rate persisted after adjustment for patient characteristics (adjusted OR, 2.10; 95% CI, 1.27-3.48; *P* = .004). The mean without-fail rate improved 14.1% more at the PREVENT sites (36.7% [58 of 158 patients] to 54.0% [95 of 176 patients], an absolute improvement of 17.3%) than at the matched control sites (38.6% [345 of 893 patients] to 41.8% [363 of 869 patients], an absolute improvement of 3.2%) (*P* = .01) (Table 3 and Figure 2).

**Table 2.** Effectiveness Comparing the Baseline Period With the Implementation Period at the PREVENT Sites^a^

Guideline-recommended process of care	Baseline (n = 162)		Implementation (n = 189)		Partially adjusted^b^		Fully adjusted^c^	
	Eligible, No.	Pass rate, No. (%)	Eligible, No.	Pass rate, No. (%)	OR (95% CI)	*P* value	OR (95% CI)	*P* value
Anticoagulation for atrial fibrillation	30	19 (63.3)	27	27 (100)	28.68 (1.79-458.94)	.02	22.54 (1.72-294.94)	.02
Antithrombotic use	142	139 (97.9)	165	159 (96.4)	0.71 (0.17-3.01)	.64	0.50 (0.08-2.95)	.44
Brain imaging	158	148 (93.7)	176	173 (98.3)	3.69 (0.97-14.07)	.06	3.95 (1.01-15.37)	.048
Carotid artery imaging	155	119 (76.8)	173	147 (85.0)	1.92 (1.06-3.47)	.03	1.99 (1.02-3.91)	.04
High- or moderate-potency statin therapy	136	92 (67.6)	152	124 (81.6)	1.99 (1.13-3.51)	.02	2.02 (0.98-4.14)	.06
Hypertension control	120	93 (77.5)	124	102 (82.3)	1.28 (0.67-2.45)	.45	1.15 (0.57-2.35)	.69
Neurological consultation	155	103 (66.5)	173	138 (79.8)	2.15 (1.25-3.71)	.006	2.09 (1.19-3.66)	.01
Without-fail rate	158	58 (36.7)	176	95 (54.0)	2.15 (1.34-3.45)	.002	2.10 (1.27-3.48)	.004

Abbreviations: OR, odds ratio; PREVENT, Protocol-Guided Rapid Evaluation of Veterans Experiencing New Transient Neurological Symptoms.

^a^ The PREVENT sites refer to the 6 participating sites that engaged in active quality improvement by implementing the intervention; 158 baseline and 176 intervention patients were eligible for at least one process of care and were included in the quality-of-care analyses.

^b^ The partially adjusted results were obtained from a model that included a random-effects size analysis for facility and fixed effects for wave.

^c^ The fully adjusted results were obtained from a model that included a random-effects size analysis for facility and fixed effects for wave and also included patient characteristics associated with each process of care measure, the consolidated measure of quality, or the without-fail rate (eTable 3 in Supplement 2 lists the specific variables included in each model).

**Table 3.** Effectiveness Comparing the Change Over Time at the PREVENT Sites vs the Matched Control Sites^a^

Guideline-recommended process of care	Matched control sites			PREVENT sites			Change in PREVENT sites vs matched control sites, %^c^	Unadjusted			Adjusted		
	Pass/eligible, %		Absolute difference, %^b^	Pass/eligible, %		Absolute difference, %^b^		OR (95% CI)		*P* value for interaction	OR (95% CI)		*P* value for interaction
	Baseline (n = 973)	Implementation (n = 968)		Baseline (n = 162)	Implementation (n = 189)			Matched control sites	PREVENT sites		Matched control sites	PREVENT sites	
Anticoagulation for atrial fibrillation	95/127 (74.8)	106/141 (75.2)	+0.4	19/30 (63.3)	27/27 (100)	+36.7	+36.3	1.03 (0.59 to 1.78)	32.84 (1.76 to 612.27)	.02	1.48 (0.77 to 2.86)	24.54 (1.24 to 484.85)	.07
Antithrombotic use	746/792 (94.2)	750/799 (93.9)	−0.3	139/142 (97.9)	159/165 (96.4)	−1.5	−1.2	0.95 (0.62 to 1.44)	0.58 (0.14 to 2.40)	.52	1.52 (0.87 to 2.63)	0.34 (0.06 to 1.84)	.10
Brain imaging	828/877 (94.4)	807/852 (94.7)	+0.3	148/158 (93.7)	173/176 (98.3)	+4.6	+4.3	1.07 (0.70 to 1.63)	3.82 (1.02 to 14.34)	.07	1.10 (0.72 to 1.68)	3.98 (1.05 to 15.11)	.07
Carotid artery imaging	641/849 (75.5)	653/841 (77.6)	+2.1	119/155 (76.8)	147/173 (85.0)	+8.2	+6.1	1.04 (0.82 to 1.31)	1.80 (1.00 to 3.23)	.09	1.02 (0.79 to 1.32)	1.86 (0.98 to 3.53)	.09
High- or moderate-potency statin therapy	478/727 (65.7)	508/725 (70.1)	+4.4	92/136 (67.6)	124/152 (81.6)	+14.0	+9.6	1.18 (0.94 to 1.48)	2.04 (1.17 to 3.58)	.08	1.12 (0.86 to 1.48)	1.99 (1.02 to 3.87)	.12
Hypertension control	468/620 (75.5)	485/649 (74.7)	−0.8	93/120 (77.5)	102/124 (82.3)	+4.8	+5.6	0.93 (0.72 to 1.21)	1.26 (0.67 to 2.40)	.39	0.97 (0.74 to 1.28)	1.17 (0.59 to 2.30)	.63
Neurological consultation	627/850 (73.8)	675/843 (80.1)	+6.3	103/155 (66.5)	138/173 (79.8)	+13.3	+7.0	1.42 (1.08 to 1.88)	2.16 (1.25 to 3.75)	.19	1.39 (1.04 to 1.86)	2.10 (1.18 to 3.73)	.21
Without-fail rate	345/893 (38.6)	363/869 (41.8)	+3.2	58/158 (36.7)	95/176 (54.0)	+17.3	+14.1	1.07 (0.88 to 1.30)	2.12 (1.34 to 3.38)	.008	1.06 (0.85 to 1.33)	2.11 (1.28 to 3.47)	.01

Abbreviation: OR, odds ratio; PREVENT, Protocol-Guided Rapid Evaluation of Veterans Experiencing New Transient Neurological Symptoms.

^a^ The PREVENT sites refer to the 6 participating sites that engaged in active quality improvement by implementing the intervention. Six control sites were matched to each PREVENT site on the basis of transient ischemic attack patient volume, facility complexity (ie, teaching status, intensive care unit level), and quality of care (measured by the without-fail rate).

^b^ The absolute difference was calculated as the difference in the process of care between the intervention period and the baseline period; therefore, a positive change indicates improved performance, and a negative change indicates a decrement in performance over time.

^c^ The change in performance (difference of differences) was calculated as the absolute change in quality at the PREVENT sites minus the absolute change at the matched control sites; therefore, a positive number indicates greater improvement at the PREVENT sites.

The mean (SD) consolidated measure of quality (secondary outcome) also improved at the PREVENT sites, from 79.6% (20.1%) to 88.0% (15.9%) (*P* < .001). Similarly, the improvement in the consolidated measure of quality was greater at the PREVENT sites (from 79.6% to 88.0%, an absolute improvement of 8.4%) than at the matched control sites (from 80.4% to 82.6%, an absolute improvement of 2.2%) (*P* = .01). At the PREVENT sites, the observed pass rates increased for 6 of the 7 primary processes of care from baseline to active implementation; however, few of the improvements were statistically significant after adjustment (Table 3).

At the PREVENT sites, the observed 90-day all-cause mortality rate decreased from 2.5% (4 of 162 patients) to 1.6% (3 of 189 patients), a decrement of −0.9%; at the matched control sites, the observed 90-day mortality rate similarly declined from 2.3% (22 of 973 patients) to 1.7% (16 of 968 patients), a decrement of −0.6% (eTable 6 in Supplement 2). Similarly, the 90-day stroke rate, the combined 90-day stroke or death rate, and the recurrent event rate decreased from the baseline period to the implementation period; although the decrements were modestly higher for the PREVENT sites than for the matched control sites for all-cause mortality, stroke, and stroke or death, none of these differences were statistically significant (eTable 6 and eFigure 1 in Supplement 2).

### Sensitivity Analysis

The results of the sensitivity analysis that included all patients with TIA (168 patients at baseline, 196 patients after active implementation) were identical to the results obtained in the main analysis, which included patients with a first TIA during the study period (162 patients at baseline, 189 patients after active implementation). The results of the sensitivity analyses that excluded patients 90 years or older (155 patients at baseline, 182 patients after active implementation) were likewise similar to the main results.

## Discussion

These results demonstrate that a multifaceted QI intervention was associated with improved quality of TIA care across diverse hospitals and clinical teams; specifically, we observed a net improvement of 14.1% at PREVENT sites compared with matched control sites. Our results contribute to the growing literature demonstrating that improvements in cerebrovascular disease quality of care are achievable with complex interventions that are implemented by clinical staff who are committed to engaging in QI.^39^ Previous studies^23,39,40^ seeking to improve quality and timeliness of TIA care have varied in terms of the targeted population (stroke, TIA), details of the QI intervention, and targeted processes of care; however, common intervention components have included audit and feedback in combination with external facilitation to support clinical teams, staff and patient education, checklists or clinical pathways, and electronic tools. Despite the heterogeneity in design, the QI interventions in general have successfully improved processes of care. For example, Machline-Carrion et al^39^ implemented a QI program seeking to improve care for patients with ischemic stroke and TIA and found that the all-or-none measure of quality was higher among patients in the intervention hospitals (49.2%) than in the control hospitals (25.2%) (OR, 2.59; 95% CI, 1.22-5.53; *P* = .01), a difference of 24.0%. Although the study by Machline-Carrion et al^39^ differed substantially from the present study (eg, included patients with stroke and focused on acute stroke processes of care like thrombolytics), the finding that the all-or-none measure varied between 25.2% and 49.2%, whereas the consolidated measure of quality varied between 77.8% and 85.3%, is similar to the present study, where the without-fail rate varied between 36.7% and 54.0% and the consolidated measure of quality varied between 79.6% and 88.0%. Although a facility may be satisfied with an 88.0% pass rate on a consolidated measure of quality, at the patient-level, only 54.0% of patients at that facility received all of the care for which they were eligible. In a study^30^ of patients with TIA and ischemic stroke, without-fail care was associated with 31.2% lower odds of 1-year mortality (adjusted OR, 0.69; 95% CI, 0.55-0.87), providing evidence to support the association between quality and outcome. Health care systems should consider including TIA in their existing stroke quality measurement programs and should consider evaluating progress against all-or-none quality measures.

The without-fail rate can be a difficult measure to improve. Patients pass the without-fail rate if they received all of the processes of care for which they were eligible (eFigure 2 in Supplement 2 shows the calculation of the without-fail rate). Consider 2 hypothetical facilities, each with a without-fail rate of 40%. In 1 case, the quality problems span several processes of care (eFigure 2 in Supplement 2). At this hypothetical facility, the QI team would have had to improve a broad range of processes to ensure that all patients received all of the care for which they were eligible. This scenario was the case at 1 PREVENT site, where the QI team implemented comprehensive systems of care to address the multiple areas in which quality gaps were evident, including routine monitoring of quality data, prospective real-time interventions to address gaps in care, implementation of new order sets, and facility-wide education programs. It took time to implement these activities; hence, the without-fail rate improved during the 1-year implementation period (from 16% to 35%), but did not achieve the goal of at least 50% that the team established for themselves at the beginning of the project. However, we hypothesize that the without-fail rate will continue to improve at this facility in the postimplementation period given that TIA quality of care management has been imbedded in routine operations. Consider a second hypothetical facility at which the without-fail rate is 40% but where patients with TIA received all of the measures for which they were eligible except for problems in 1 process (eFigure 2 in Supplement 2). At this second facility, the QI team could focus on a single process of care. This scenario was the case at another PREVENT site, which achieved substantial improvements in the without-fail rate (from 39% to 60%) by improving the prescription of high- or moderate-potency statin therapy for eligible patients. However, given that the team at this site did not implement any facility-wide modifications in procedures, nor did they establish routines to monitor quality of care data, we hypothesize that their quality will diminish during the postimplementation period.

### Strengths and Limitations

A methodological strength of the present study was the augmentation of the cluster design with the use of the matched control comparison. A limitation of nonrandomized cluster design in cases where either the baseline period is short or the sample size during the baseline period is insufficient to allow for segmentation of the baseline period into distinct subperiods can be the potential for confounding by temporal trends.^41,42^ The improvement observed at the matched control sites suggests that the quality of care improved over time and confirms the importance of evaluating care at the matched control sites.

The PREVENT QI program is an example of a learning health care system in action.^15^ Learning health care systems develop QI programs that are data driven, meet the needs of stakeholders, and dynamically adapt to changes in performance and context. For example, 3 processes of care with the greatest opportunities for improvement for the greatest number of eligible patients at baseline included neurological consultation, hypertension control, and the use of high- or moderate-potency statin therapy. Although the without-fail rate included 7 processes of care, the 3 processes listed above were common QI targets at the PREVENT sites, demonstrating how QI programs can be data driven and adaptable. Both hypertension control and the use of high- or moderate-potency statin therapy are part of the scope of practice of existing VA pharmacy staff; all of the PREVENT sites engaged pharmacists on their QI teams, providing an example of how a QI project can leverage local resources.^43^ Another common approach was to increase the proportion of patients with TIA who were admitted given the observation that patients who were discharged from the ED were less likely to receive guideline-recommended processes of care, providing an example of using data infrastructure to identify strategies for improvement.^12^

This study has some limitations. First, the primary limitation is that the PREVENT program was implemented only within VA facilities, which may limit generalizability; future research should evaluate its effectiveness when implemented at non-VA sites, where the health care infrastructure (eg, electronic health record and QI culture) may vary. Moreover, given that the sites were selected on the basis of gaps in quality of care at baseline, the results may not generalize to high-performing hospitals. Second, there may be some diagnostic uncertainty when making a diagnosis of TIA.^44^ However, given that miscoding is likely to exist across all of the sites, it is unlikely that this limitation altered the assessment of the intervention association. Third, because the intervention included multiple components, we are unable to isolate and estimate the unique associations of each specific element. Fourth, neither site selection nor allocation to waves was randomized. Therefore, the possibility of selection bias cannot be eliminated; in addition, although the matched control sites were similar to the PREVENT sites in several key aspects, they were not matched on the unmeasurable characteristics associated with motivation to improve TIA care. The results should be considered as associations and not causal relationships. Fifth, the anticoagulation for atrial fibrillation measure evaluated whether anticoagulation was prescribed for patients with a diagnosis of atrial fibrillation (preexisting or new diagnosis); the measure did not evaluate whether patients received screening for atrial fibrillation. Sixth, we cannot explain why modest decrements in pass rates for antithrombotic use were observed both at the PREVENT sites and the matched control sites (Table 3); future studies should explore potential mechanisms of this finding. Seventh, although a 6-site sample provided adequate power for the detection of changes in processes of care, the study was not powered to detect changes in patient outcomes. Future studies might include a larger number of facilities and more patients to provide power to detect differences in patient outcomes, such as recurrent stroke or mortality. Research is ongoing to examine the sustainability of TIA QI at the PREVENT sites.

## Conclusions

We believe that the PREVENT sites achieved remarkable improvements in quality of care despite heterogeneity in their baseline quality of care and team composition. Other health care systems interested in improving TIA quality of care may consider making similar interventions available to their clinical teams, which include readily available implementation tools, professional educational materials, an audit and feedback mechanism, external facilitation support, and virtual collaborative learning. The VA was the first US federal agency to support the core values of the learning health care system, which has been recognized as an exemplary organization that harnesses the power of data to improve the health of the populations it serves.^45^ Based on the observed improvements in quality of care, the PREVENT QI program was deployed nationwide across the VA health care system in 2019.

## Notes

Supplement 1. **Trial Protocol**

Supplement 2. **eTable 1.** Definitions of Seven Processes of Care Included in the Without-Fail Measure **eTable 2.** Diagnosis Codes **eTable 3.** Risk Adjustment Variables for Each Process Measure or Outcome **eTable 4.** Facility Staffing for Matched Control and PREVENT Sites **eTable 5.** Without Fail Rates by Facility and Study Period **eTable 6.** Patient Outcomes at PREVENT Sites and Matched Control Sites **eFigure 1.** Time to Recurrent Events: Disease-Free Survival **eFigure 2.** Illustration of the Without-Fail Rate for Two Hypothetical Facilities

Supplement 3. **Data Sharing Statement**
