# Supplementary material for: Assessment of the Protocol-Guided Rapid Evaluation of Veterans Experiencing New Transient Neurological Symptoms (PREVENT) Program for Improving Quality of Care for Transient Ischemic Attack: A Nonrandomized Cluster Trial
Source: JAMA Netw Open. 2020 Sep 8;3(9):e2015920. doi: 10.1001/jamanetworkopen.2020.15920 (PMC7489850; doi:10.1001/jamanetworkopen.2020.15920)
Supplement: Supplement 1. — Trial Protocol [file jamanetwopen-e2015920-s001.pdf]

Protocol-Guided Rapid Evaluation of Veterans Experiencing New Transient Neurological Symptoms (PREVENT)

ANALYSIS PLAN

1. BACKGROUND

Approximately 8,500 Veterans with transient ischemic attack (TIA) or stroke are cared for in a Department of Veterans Affairs (VA) Emergency Department (ED) or inpatient ward annually.[1] Patients with transient ischemic attack (TIA) are at very high risk of recurrent vascular events; over the long-term 11% per year will have a stroke, myocardial infarction or vascular death.[2-4] Because patients with TIA and minor stroke have little neurological impairment yet are at high risk of recurrent events, including disabling stroke, they are ideal candidates for interventions to reduce their risk of adverse events. Several studies have demonstrated that interventions which deliver timely care reduce the risk of recurrent vascular events by 70%.[5]

Our group has assessed the quality of care for Veterans with TIA and minor stroke cared nationwide and has found considerable gaps in care quality (e.g., 51% of eligible Veterans receive carotid imaging, 27% receive indicated antihypertensive medication intensification).[6] In other words, Veterans are not receiving the timely, guideline concordant care they need to prevent recurrent vascular events. The objective of the PREVENT project is to develop and evaluate an intervention program to improve the quality of care for Veterans with TIA and minor stroke.

2. SPECIFIC AIMS

Aim 1. To develop a program to improve the care of Veterans with TIA or minor stroke that can be deployed nationwide. The program includes 5 components: (1) a reporting system that is based on validated electronic quality measures that allow staff to monitor the time-sensitive processes of care and outcomes of their Veterans with TIA; (2) clinical programs based on existing VHA infrastructure (e.g., a pharmacist-based TIA medication management program); (3) a staff education and training program; (4) protocols and templates for use by clinicians, nurses and pharmacists; and (5) quality improvement support including a virtual collaborative to share lessons learned across sites. We will measure users' assessment of the PREVENT program in terms of usability, complexity, and relative advantage to deliver and improve care to Veterans with a recent TIA or minor stroke. We hypothesize that we can develop and facilitate the implementation of a program to improve guideline concordant care for Veterans with a recent TIA or minor stroke that achieves a high degree of staff satisfaction.

Aim 2. To evaluate the effectiveness of an intervention program for Veterans with TIA or minor stroke against usual care. Teams at the 6 intervention sites will be given access to the intervention program in 3 waves of 2 sites per wave. The primary effectiveness outcome is the proportion of Veterans who received all of the guideline-concordant processes of care for which they are eligible referred to as the "Without-Fail" care rate. We will compare the Without-Fail rate observed in the post-implementation phase to the baseline period. We hypothesize that the Without-Fail care rate will be improved by the intervention program.

Aim 3. To evaluate the implementation of the intervention program across the 6 participating sites. The two primary implementation outcomes will be the number of implementation activities completed during the one-year active implementation period and the final level of team organization (defined as the Group Organization (GO Score)) for providing and improving TIA care at the end of the 12-month active implementation period. A covariate will include the

degree of participation [Total # of calls attended/12 active implementation calls] in the monthly collaborative calls as noted in the call attendance. Implementation activities completed will be identified from multiple data sources: key updates and topics discussed during the collaborative calls; monthly action plan achievements and additional facilitator notes; the HUB implementation plan updates; and 6 and 12-month interviews. We hypothesize that sites that engage in ongoing [positive] implementation strategies of reflecting and evaluating, goal setting, and planning will realize the greatest implementation success (defined across the two primary implementation outcomes: number of implementation activities and the Group Organization (GO score)). We also hypothesize that sites with greatest implementation success will achieve the highest Without-Fail rate. We further plan to map implementation activities completed onto the key implementation strategies (reflecting and evaluating, goal setting, and planning) stratified by valence [positive & negative] based upon the mixed methodology coding.

Secondary Aim To evaluate the sustainability of the program. Sustainability will be evaluated over a one-year period that begins after the one-year active implementation period. We will compare the Without-Fail rate in the sustainability period to the post-implementation period. Our hypothesis is that sites that implement a facility-wide, TIA System of Care (reflected in a final 12-month GO score between 8-10) will demonstrate the greatest program sustainability as measured by the Without-Fail rate. Further, sites that maintain their implementation activities involving PREVENT-related program elements during the sustainability period will demonstrate the greatest program sustainability as measured by the Without-Fail rate.

3. RATIONALE

Imperative to Provide Timely Care for Patients with TIA and Minor Stroke. Because the risk of recurrent vascular events is highest early after an index cerebrovascular event,[4] preventive actions must be applied early to maximize the benefit. Several non-VA studies have demonstrated improved outcomes for TIA/minor stroke patients with programs that emphasize early evaluation and management including: lower stroke rate, reduced lengths of hospital stay, reduced costs, and improved vascular risk factor management.[7-9] The EXPRESS study demonstrated that early assessment and management of TIA/minor stroke patients reduced the 90-day stroke risk by 80% (from 10.3% to 2.1%).[9] Similarly, the SOS-TIA study, found a 79% reduction in the 90-days stroke rate (from a predicted rate of 5.96% to an observed rate of 1.24%).[10] The FASTEST[11] trial demonstrated that an electronic protocol for use in general practice improved care and outcomes for patients at lower cost than usual care; guideline adherence improved from 41% to 76% and the 90-day recurrent vascular event rate decreased from 11.9% to 3.5%.[11] FASTEST is particularly relevant because it integrated care across settings and was designed to improve care for patients with limited access to specialists.[11]

Strength of the Evidence Supporting the Intervention. Substantial evidence exists related to the design of our proposed intervention and the focus on specific processes of care. First, FASTER, SOS-TIA and EXPRESS all included algorithms or protocols that facilitate the timely delivery of care. In addition, numerous studies have demonstrated that nurse-administered, telephone-delivered care can improve vascular risk factor control for Veterans.[12,13] The planned intervention similarly includes algorithms and protocols for the timely delivery of guideline concordant care. Finally, the processes of care that are the focus of the intervention are all supported by the AHA/ASA secondary prevention guidelines or other relevant professional organization guidelines.[14] For each process in this project, evidence supports the association between the process of care and improved outcomes. Moreover, most of the processes were included in one of the prior trials that have demonstrated that protocol-based care dramatically reduces the risk of recurrent vascular events.[11,15]

4. METHODS

4.1 Aim 1 Methods (Program Development)

The development of the PREVENT program was based on four main components: (1) interviews with VHA staff members involved in the care of patients with cerebrovascular disease;[16] (2) validation of electronic quality measures against chart review for use in ongoing assessment of a broad range of processes of care at all VHA medical centers;[17] (3) baseline quality of care data identifying potential gaps in care that should serve as targets for quality improvement;[18] and (4) the existing literature regarding effective TIA clinical programs. [11-13,15]

As part of a prior project,[16] we met with diverse staff members involved in the care of patients with stroke and TIA including: stroke team members (which included nursing, neurology, and ED members), pharmacy, primary care providers, hospitalists, radiology, vascular surgery, cardiology, ophthalmology, systems redesign, and quality management staff. Based on interviews with these staff, we identified barriers to providing high quality TIA care including: gaps in knowledge; lack of performance data; uncertainty about how to engage in quality improvement; inadequate care coordination; and information technology barriers.[16] The PREVENT intervention was designed to address each of these barriers; to utilize existing VHA resources to improve care for areas with demonstrated gaps in quality; and to include strategies which were successfully deployed in prior TIA intervention studies.

PREVENT Intervention Components Description

The PREVENT QI program includes 5 components: (1) a reporting system that is based on validated electronic quality measures that allow staff to monitor the time-sensitive processes of care and outcomes of their Veterans with TIA; (2) clinical programs based on existing VHA infrastructure (e.g., a pharmacist-based TIA medication management program); (3) a staff training program; (4) protocols and templates for use by clinicians, nurses and pharmacists; and (5) quality improvement support including a virtual collaborative to share lessons learned across sites.

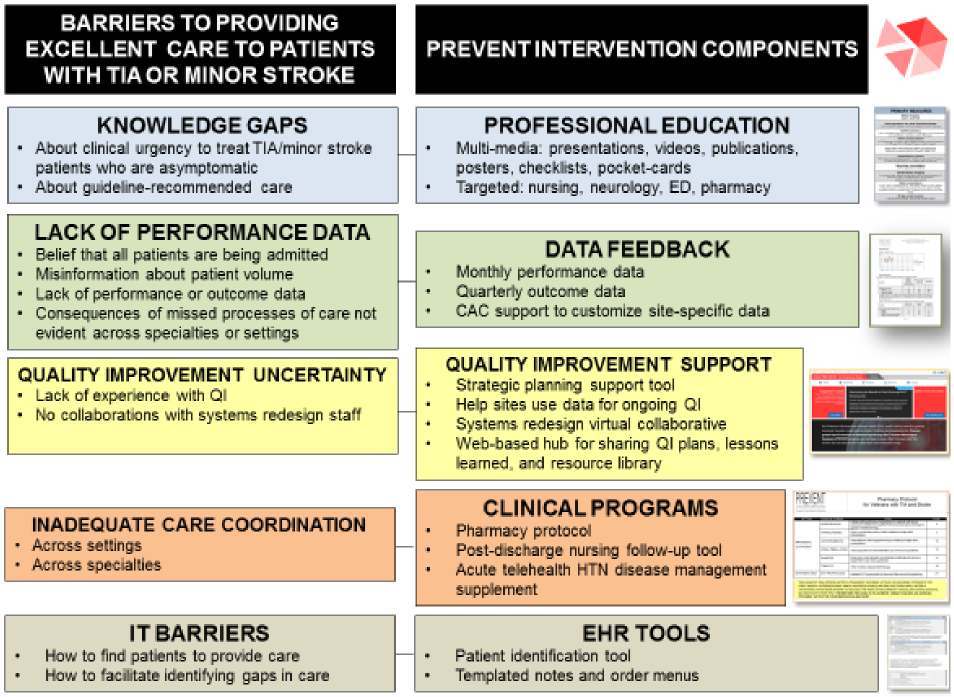

Implementation Strategies
The PREVENT program employs three primary implementation strategies: (1) team activation via (a) audit and feedback, (b) reflecting and evaluating, (c) planning, and (d) goal setting; (2) external facilitation; and (3) building a community of practice. In addition, PREVENT allows for local adaptation of the intervention and also takes advantage of peer pressure while providing facilitation support to the site champion. During the active implementation period, we are tracking the adopted and adapted PREVENT program elements chosen by each site across the waves. Adoption is defined as PREVENT program elements chosen to be implemented at a local site. Adaptation is defined as site specific modifications made to the chosen PREVENT program elements during implementation.

Aim 1 Evaluation
Our assessment of the program development is based on the end-user evaluation of the usability, and complexity of the program to deliver and to improve care to Veterans with TIA.[19] We will obtain these data during interviews conducted 6-months after the start of active implementation and again at the end of active implementation.

**Aim 1 Assessments: staff satisfaction with the intervention**<br>*Obtained at 6-months and 1-year after the start of active implementation*
Participant perceptions and overall experience with each of the following elements of PREVENT as well as: the ease of use, how useful each element is to providing or improving care, and whether they are using each element:<br>1. PREVENT overall<br>2. The five components<br>3. Kick-Off<br>4. Hub (overall perceptions, usability, how are they using the hub)<br>5. Virtual collaborative/monthly calls<br>6. External facilitation
Participant perceptions of PREVENT program complexity and usability
Describe any sharing of tools with peers or with leadership at own facility, with other facilities
Which components of PREVENT are the most helpful to you as you work to improve outcomes for your Veteran patients with TIA/minor stroke?
Specific quality improvement activities planned for next 6 months

Audience-response system (ARS) feedback about program components and staff written evaluations obtained during the Kick-Off will also inform the assessment of staff satisfaction with the intervention and implementation strategies.

Aim 1 Analysis
Aim 1 Hypothesis: that we can develop a program to improve guideline concordant care for Veterans with a recent TIA or minor stroke that achieves a high degree of staff satisfaction (defined as users' assessment of the PREVENT program in terms of usability, complexity, and relative advantage to deliver and improve care to Veterans with a recent TIA or minor stroke).

The assessment of staff satisfaction with the PREVENT program and its components will utilize mixed methods. We will seek to identify the components of the intervention that were considered to be most useful and/or most important to the local PREVENT program in both the

6-and 12-month interviews as well as the staff program evaluations completed at the end of the day long site kick off.

Interviews with facility stakeholders will be conducted in person during site visits or by telephone. Key stakeholders are defined as front line staff, managers, and leadership affiliated with the delivery of TIA care. We will also accept “snowball” referrals from key stakeholders. Upon receipt of verbal consent, all interviews will be audio recorded. The audio recordings will be transcribed verbatim. All transcripts will be de-identified and imported into an Nvivo11 project file for data coding and analysis. Project team members will independently read and code identical transcripts using a common codebook derived from the semi-structured interview guide and project analysis plan. We plan to code for the presence or absence of constructs as well as the magnitude and valence each specified Consolidated Framework for Implementation Research (CFIR) constructs. Each coded transcript will be merged into a single file, and the project team will meet as a group to review and discuss similarities and differences in the coding selections until a shared understanding of each item in the codebook had been developed. All codes will be defined with examples in a common codebook and updated as needed by team agreements on modifications. At a minimum, two coders per interview will be assigned to each interview to best capture the specified constructs. Completed coded interviews from each site will be merged into Facility level estates and ultimately, all Facility level estates will be merged into a Project Universe file for analysis.

<u>Coding for Usability and Complexity</u>. We will derive the users’ assessment of the intervention using the Intervention Characteristics domain from the Consolidated Framework for Implementation Research (CFIR). We will code statements on the presence of Intervention specific CFIR constructs about how the PREVENT program was useful for their local adopted program to delivery TIA care as well as statements on how it was not helpful; we will code statements about how complex the PREVENT program components were to implement as well as statements on the lack of complexity/ease of the components’ implementation to improve the 7-key metrics of TIA related care.

<u>Coding for Context</u>. We will continue to use the CFIR coding methodology to denote the presence or absence of the local context including the following from the inner setting: structural characteristics, networks and communications, culture, learning climate, leadership engagement, and available resources as well as champions in the implementation process.

<u>Key Implementation Strategies and CFIR constructs</u>. We will expand the CFIR coding to include valence (positive + and negative -) influences on implementation for our key implementation strategies: reflecting and evaluation; goal setting and feedback; planning; and champions.

4.2 Aim 2 Methods (Effectiveness)

Design

This five-year stepped-wedge[20,21] evaluation includes a total of 6 sites, where active implementation is initiated in 3 waves each of which includes 2 sites. The project involves three phases at each of the six sites: a one year-baseline phase, a one-year active implementation phase, and a sustainability phase of at least one year in duration follows the end of active implementation.

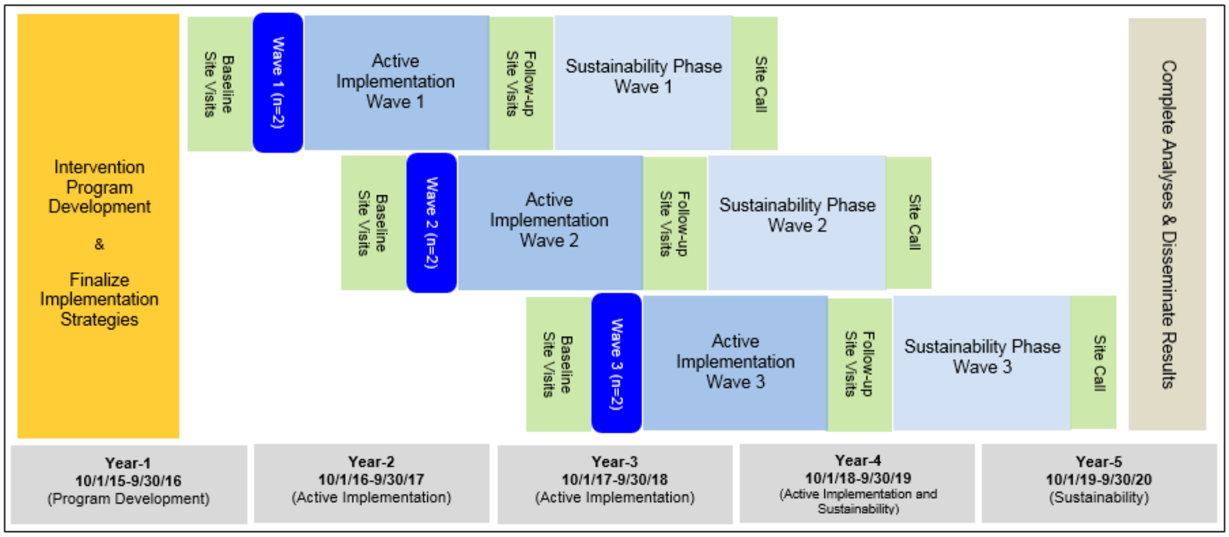

Power and Sample Size

The six site, stepped wedge design provides >90% power to detect an improvement in the mean facility Without-Fail rate from 25% during the baseline data period to >50% during the active implementation data period. The goal for the sample size was to recruit sites with at least 50 TIA patients per year, however, power was preserved with at least 30 TIA patients per year.

The figures below provide the plots of the power calculated based on the modified formula for testing the intervention effect $H_0: \theta = 0, H_1: \theta = \theta^M$ for the intervention effect size θ^M ranging from 0.1 to 0.3 when taking account for a potential decrement in intervention effect over time γ. The plot was based on a total of 6 clusters with a cluster sample size of 30, 50 and 70, respectively. The results demonstrated that this variant of SWD generally has a reasonable power (greater than or equal to 0.90) for detecting the intervention effect when the effect size is at least 0.15 with a cluster size no less than 30. The coefficient of variation (CV) was set at 0.2, 0.4, 0.6, and 0.8 to cover a wide range of the between-hospital variation. The CV seemed to have little effect on power.

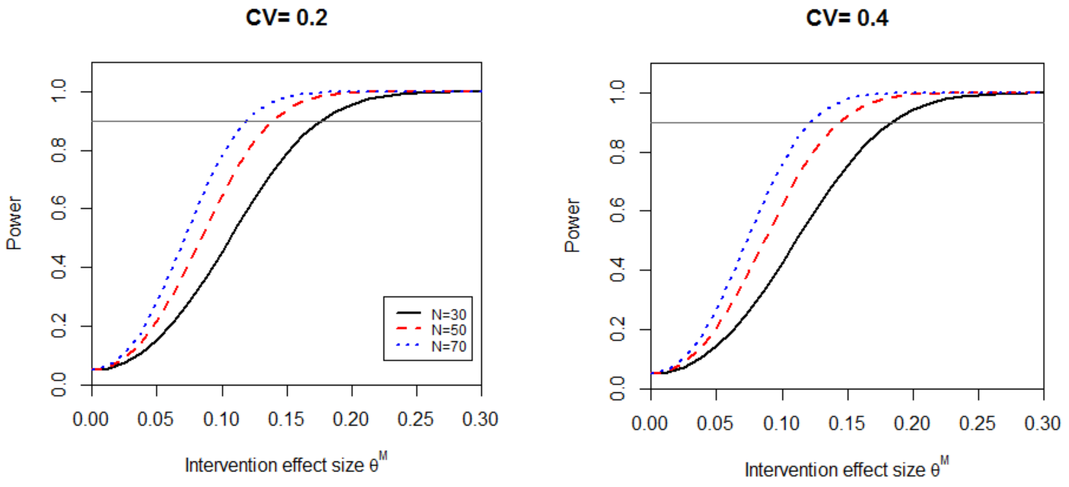

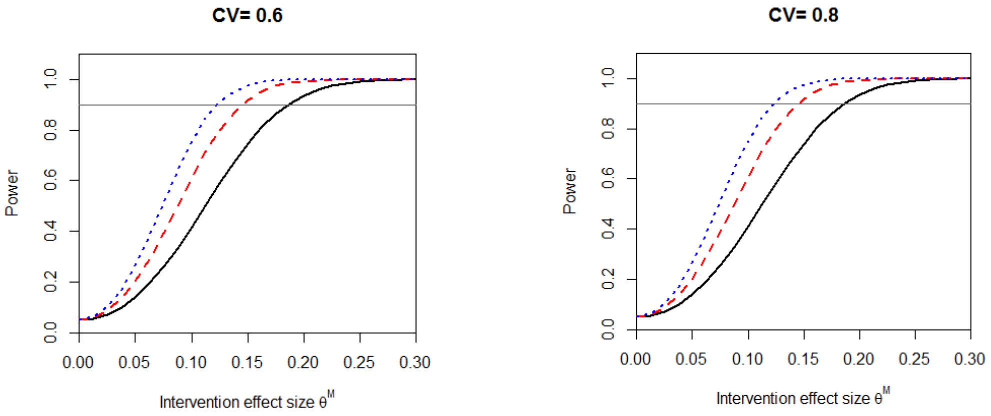

Site Selection
Sites were invited to participate on the basis the basis of demonstrated gaps in quality of care (identified using the validated eQMs). Specifically, sites were invited to participate if they had baseline without-fail rates of <50%. Invitations were sent via email to higher volume sites first and recruitment continued until 6 participating sites were identified. No minimum threshold on volume was employed because our goal was to include sites which were diverse so that the results might be widely generalizable across the full spectrum of VHA facilities.

Allocation of Sites to Waves
Although some stepped-wedge trials randomly assign facilities to waves, PREVENT sites were allocated to waves on the basis of the pragmatics of the ability to schedule baseline and Kick-Off meetings.

Intervention Implementation
The multi-faceted PREVENT intervention (described above) will be implemented via a Kick-Off meeting which includes all relevant staff members at participating sites and then through monthly telephone conference calls which include all members of the teams at the participating sites after the Kick-Off. Participants in the collaborative calls receive CME (Continuing Medical Education) for clinicians and pharmacists or CEU (Continuing Education Units) for nurses. All data, quality improvement plans, professional education materials and other resources will be shared with the sites through the web-based portal, referred to as the PREVENT hub.

Focus on Staff/Facilities
The intervention targets participating site staff members. Individual patients are <u>not</u> the target of the intervention or the implementation activities.

Effectiveness Outcome: The Without-Fail Rate
The primary effectiveness outcome, referred to as the Without-Fail care rate, is the proportion of Veterans who received all of the care for which they are eligible from among 7 key processes of care: brain imaging, carotid artery imaging, neurology consultation, hypertension control, anticoagulation for atrial fibrillation, antithrombotics, and high/moderate potency statins. The Without-Fail rate is calculated at the facility level based on electronic health record data using validated algorithms.[17] Data from all TIA patients cared for in the participating facilities'

Emergency Departments (ED) or inpatient settings are included in the measurement of the without-fail rate.

Effectiveness Data Period Definitions

Sites	Baseline Data Period (Control Period)	Date of Baseline Site Visit	Pre-implementation Data Period	Date of Kick-Off	Post-Implementation Data Period	Sustainability Data Period
	1 year before the baseline visit		*Time between baseline visit and Kick-Off*		*1 year after Kick-Off: Months 2-13 after the Kick-Off*	*Variable period: Months 14 after the Kick-Off until 9/30/19*
A	8/21/15-8/21/16	8/22-23/16	8/24/16-7/10/17	7/11/2017	8/11/17-8/10/18	8/11/18-9/30/19
B	8/29/15-8/29/16	8/30-9/1/16	9/2/16-7/25/17	7/26/2017	8/26/17-8/25/18	8/26/18-9/30/19
C	7/30/16-7/31/17	8/1-2/17	8/3/17-10/17/17	10/18/2017	11/18/17-11/17/18	11/18/18-9/30/19
D	6/12/16-6/12/17	6/13-14/17	6/15/17-11/1/17	11/2/2017	12/2/17-12/1/18	12/2/18-9/30/19
E	8/23/16-8/23/17	8/24-25/17	8/26/17-12/6/17	12/7/2017	1/7/18-1/6/19	1/7/19-9/30/19
F	1/30/2017-1/30/2018	1/31-2/1/18	2/2/2018-4/12/2018	4/13/18	5/13/18-5/12/19	5/13/19-9/30/19

Primary Aim 2 Analysis:

Descriptive Analysis: The Without-Fail care rate will be estimated at each site for each of the three periods (baseline, post-implementation, and sustainability) to exam the temporal trends in the intervention effectiveness. The 95% confidence intervals for the rates will be provided.

Statistical Models: Due to the variation of sample size across the sites and across the implementation phases, generalized mixed-effects models (GLMM) at patient level with multilevel hierarchical random effects will be developed to analyze the intervention effects on the Without-Fail care rate during the post-implementation period and sustainability period compared with the baseline period.

The baseline data period is the one-year prior to the date of the baseline site visit. The active implementation phase data period extends for one year from month 2-13 after the Kick-Off event. For the primary effectiveness analysis, the main comparison is the mean facility Without-Fail rate across the six sites during the baseline data (control) period (blue shading above) versus the mean facility Without-Fail rate across the six sites during the post-implementation data period including the active implementation and one year after the active implementation phases (green and pink shadings above). We propose to model the "Without-Fail" care rate by

$$log \frac{P(Y_{ij,k} = 1|X_{ij,k}, Z_{ij,k}, W_{ij,k})}{1 - P(Y_{ij,k} = 1|X_{ij,k}, Z_{ij,k}, W_{ij,k})} = \mu + \alpha_i + \beta_j + X_{ij,k}\theta + Z_{ij,k}\gamma + W_{ij,k}^t\tau$$

where i indexes the site ($i = 1,2,3,4,5,6$); j the implementation phase (1: baseline period; 2: active implementation phase; 3: one year after the active implementation phase); k the encounter at the i^{th}site and the j^{th}phase ($k = 1,2, \dots, n_{ij}$); $n_{ij}$is the total number of encounters

at the i^{th}site and the j^{th}phase; $X_{ij,k}$and $Z_{ij,k}$ the indicators of the active implementation phase and one year after the active implementation phase, respectively; $W_{ij,k}$the vector of the kth encounter's characteristics at the i^{th}site and the j^{th}phase that could include the site characteristics; $Y_{ij,k} = 1$the indicator of observation of “Without-Fail” care process on the kth encounter at the i^{th}site and the j^{th}phase. For this model, μ represents the average log odds of Without-Fail care across the 6 sites at baseline; θ the log odds ratio between the active implementation phase and the baseline indicating the intervention effect during the active implementation phase; and γ the log odds ratio between one year after the active implementation phase and the baseline indicating the intervention sustainability effect post the implementation; α_i and β_j are the site and phase specific random effects that are assumed to be multivariate normal. After fitting the model, the normal theory can be applied to test the intervention effect at the active implementation phase and the intervention sustainability effect.

Secondary Aim 2 Analyses: Several secondary effectiveness analyses are planned.

Comparison to Matched Controls
Six control sites will be matched to each of the six PREVENT active implementation sites on the basis of: TIA patient volume, facility complexity (i.e., teaching status, intensive care unit level), and quality of care (measured by the without-fail rate). The total number of control sites will be 36. The definition of the baseline period (the specific start and end dates of the one-year baseline period) for each matched control site will be identical to the definition used for the PREVENT active site to which the controls are matched. We will compare changes in quality and outcomes at control sites to changes at PREVENT sites as a method for assessing temporal trends.

Changes in Individual Processes of Care
An examination of the care rate of facility process for each of the seven primary processes of care across the six sites from the baseline period to post-implementation period will be conducted using the same analytical template as outlined for the primary analysis. A single risk adjustment model will be developed for use in evaluating the seven processes of care and will focus on baseline patient characteristics as follows:

Process	Risk Adjustment Variable
Anticoagulation for atrial fibrillation	Past history of atrial fibrillation (versus new afib)<br>Use of anticoagulation prior to index event<br>PMH of GI or intracranial hemorrhage<br>Age<br>Dementia
Antithrombotics	PMH of CAD, MI<br>PMH of diabetes<br>Anti-platelet or anticoagulant prior to index
Brain imaging	PMH of stroke or TIA
Carotid artery imaging	PMH CEA or stent<br>PMH of PVD
High or moderate potency statin	Use of statin prior to index event<br>PMH of CAD, MI<br>PMH of diabetes<br>History of intracranial hemorrhage<br>LDL-cholesterol<br>Age

Hypertension control	Kidney disease, dialysis<br>PMH diabetes<br>PMH of CAD, MI<br>Antihypertensive meds before index event<br>BP before the event and at discharge<br>Homeless<br>PCP visit
Neurology consult	Neurology FTE
General	Hospice, comfort care, AMA<br>Admitted versus ED<br>Transferred into or out of our facility<br>Nights/weekends presentation

<u>90-Day Recurrent Stroke Rate</u>

(1) The 90-Day recurrent stroke rate will be estimated at each site before (baseline and pre-implementation phase) and after (active implementation phase and one year after the active implementation phase) the intervention to examine the temporal trends in intervention effectiveness for reducing the 90-day recurrent stroke rate. The 95% confidence intervals for the rates will be provided.

(2) The similar GLMM at patient level as described for the primary analysis will be developed for the incidence of 90-day recurrent stroke across the six sites and the whole study period to study the mean change of this rate. For this model, the indicator of Without Fail care at the encounter level will be incorporated into the model as the main exploratory variable to examine if the improvement of the Without Fail care rate will result in significant reduction of the 90-Day recurrent stroke rate.

<u>90-Day Mortality</u>

The analysis plan for the 90-day recurrent stroke rate (described above) will also be implemented for the 90-Day Mortality.

<u>Disease-free Survival Analysis</u>

We will examine disease-free survival over the course of 1-year after the index event. We will use Cox proportional hazards modeling, censoring patients for either death or recurrent stroke and examine the relationship between quality of care and disease-free survival.

<u>Temporal Trends in Effectiveness</u>

An examination of the mean Without-Fail rate for the baseline data period through sustainability for all non-participating VA sites with at least 10 TIA patients per year. Given that Indianapolis VAMC has served as the coordinating center, we would not include that site in the analyses.

<u>Consolidated Quality Measure</u>

A consolidated measure of quality defined as the number of passes divided by the number of eligible patients across the seven primary processes of care will be compared for baseline versus post-implementation periods using the same approach described above for the Without-Fail rate analysis.

Secondary Processes of Care
An examination of the secondary processes of care: baseline versus active implementation data periods.

Sensitivity Analyses
The primary analysis will include the first TIA event per patient during the entire study period. In sensitivity analysis, we will include (1) the first event per period and (2) all TIA events in the analysis.

4.3 Aim 3 Methods (Implementation)
Implementation is being guided by the Consolidated Framework for Implementation Research (CFIR), which serves as the project's conceptual framework. We will use a mixed methods approach to evaluate implementation. Based on data from sites visits, observations made during Kick-Offs and collaborative telephone calls, and interviews at 6-months and 12-months after active implementation, we will assess: the number of improvement initiatives completed; the level of team organization for providing and improving TIA care; the degree to which sites engage in ongoing reflecting and evaluating, goal setting, and planning; participation in the monthly collaborative calls; the local adoption and adaptability of the program over the course of the study; and contextual factors to identify optimal context associated with implementation success. The two primary implementation outcomes will be the number of implementation activities completed and the level of team organization [GO Score] for providing and improving TIA care.

**Implementation Outcome**	**Domain**	**Measurement**
Implementation Activities		• Kick-Off plans & plan updates on Hub<br>• Collaborative call updates – action plans and FAST<br>• Interviews at 6- and 12-months<br>• Facilitator tracking sheet and updates
Team activation	Overall team activation	• GO score based on interview data, reports from sites (e.g., emails), and updates from monthly collaborative calls, facilitator tracking sheet<br>• Kick-Off participation
	Audit and feedback	• Hub usage—data pages; user interviews<br>• Interviews at 6 and12 months<br>• Relevant discussion from Collaborative Calls captured on FAST<br>• Facilitator notes and tracking sheet
	Reflecting and evaluating	• Meeting as a team to discuss data: obtained from updates during monthly calls captured on FAST and 6 & 12-month interviews<br>• Facilitator notes and tracking sheet
	Planning	• Hub usage—planning tool<br>• 6 & 12-month Interview questions and any relevant input from Collaborative Calls captured on FAST.<br>• Facilitator notes and tracking sheet
	Goal setting	• Obtained from 6 and 12 month interviews<br>• Kick off WF goal

<table>
<tr><td></td><td></td><td>• Any relevant discussion from Collaborative Calls and on FAST template
• Facilitator tracking sheet and notes</td></tr>
<tr><td>External facilitation</td><td></td><td>• Dose, Temporality, Target: Tracking sheet</td></tr>
<tr><td>Community of practice</td><td rowspan="2"></td><td rowspan="2">• Participation in monthly calls
• Posting projects and other locally developed resources on the hub
• Presenting on collaborative calls or SQUINT calls
• Sharing/discussing best practices with other facilities outside of collaborative calls [6 and 12-month interviews; Facilitator notes and tracking sheet</td></tr>
<tr><td>Cosmopolitanism</td></tr>
<tr><td>Local adaptation</td><td></td><td>• A site-specific PREVENT program description of adopted elements developed based on updates during monthly calls and this will be reviewed during interview with sites after active implementation. Adaptation based upon program contents, settings, temporality, and roles.</td></tr>
<tr><td>Peer pressure</td><td></td><td>• Evaluated during debriefs from Kick-Offs and after monthly conference calls and at the end of active implementation interviews
• FAST template recording from calls</td></tr>
<tr><td>Clinical champion</td><td></td><td>• CHAMP measure at baseline and end of active implementation interviews</td></tr>
<tr><td>Culture</td><td></td><td>• OCAI measured at baseline and end of active implementation interviews</td></tr>
</table>

GO Score – Group Organization Score[22] for Providing and Improving TIA Care is a measure of team activation on a 1-10 scale for either improving or providing TIA Care based on specified provider practices sustained at a given time period. A score between 1-3 denotes a beginning level with no facility wide approach. A score between 4-5 reflects a developing approach. A score of 6-7 denotes a basic proficiency and a score of 8 indicates an intermediate proficiency. Finally, a score of 9-10 reflects a TIA system that is implemented facility wide and can sustain key personnel turnover**.**

FAST – Fast Analysis & Synthesis Template. This template serves as a rapid, systematic method for extracting key concepts across data sources and is derived from A. Hamilton methodology[23] and used most recently to summarize qualitative findings across regions (HSRD Summer 2017 featured presentation). In a similar manner, we will utilize the FAST template to capture key concepts from the Collaborative Calls, Stakeholder interviews, and other data sources in a condensed manner that accords with interview guides**.**

Aim 3 Primary Implementation Analysis

As described above, interviews will be audio-recorded. Audio recordings will be transcribed verbatim and transcripts will be de-identified and imported into an Nvivo11 project file for data coding and analysis. Project team members will independently read and coded identical transcripts using a common codebook derived from the semi-structured interview guide. Each coded transcript will be merged into a single file, and the project team will meet as a group to review and discuss similarities and differences in the coding selections until a shared understanding of each item in the codebook had been developed.

In addition to qualitative coding, the analysis team will directly apply implementation science constructs to the qualitative data. Individual members of the analysis team will rate interview transcripts first for construct and valence (i.e., positive, neutral or negative).[24,25] When all the interviews in a site visit have been rated, the analysis team will review those ratings and then score each VAMC with a facility-level valence (i.e., positive, neutral, or negative) for the key implementation strategies and CFIR constructs: reflecting and evaluating, goal setting and feedback, planning, and champions.[24-26]

CFIR. From the 5 CFIR Domains, we are interested in the local implementation of the novel Intervention domain within the Inner Setting, i.e., each of the VAMCs. Within the Implementation Processes domain, we are interested in the clinical provider self-efficacy (Individual Domain) to implement PREVENT and to perform the 7 clinical processes upon which the without fail rate is calculated, and the organization’s cosmopolitanism (External Setting), the degree to which an organization is networked with other external organizations. The table below offers examples of how the CFIR construct for “Champions” has been coded, rated, and scored.

Example of Valence Ratings.

**Champions** (Process)		**Definition**: Individuals who dedicate themselves to supporting, marketing, and driving through an implementation, overcoming indifference or resistance that the intervention may provoke in an organization.	
	**Positive**	**Neutral**	**Negative**
**Examples**	• Presence of multiple champions in different disciplines that communicate regularly<br>• Individuals that don’t take “No” for answer; constantly talk to others about stroke<br>• Clinicians with deep, sustained interest in ACS	• Potential stroke champions have been identified, but impact is limited/limited<br>• Respondents identify champions but in ambiguous or neutral manner.	• The absence of champions has a detrimental effect on ACS<br>• Individuals may be having negative roles or stymying improvement efforts<br>• “Anti”-champions exist
**Sample Quotes**	*My motto is that if the rules weren't made to be broken, they're made to be bent, and I certainly bend a lot of them.* (1000SV2, +2)<br><br>*Wherever I am, I'm talking about stroke. If I'm in the beauty shop and saying, 'Hey you know the warning signs of stroke?' (900SV2, +2)*<br><br>*We have a team of stroke champions, multidisciplinary, most of which originated from the workgroup that developed the care path. We meet weekly to run through our stroke patients for that week and kind of do Multidisciplinary Team*	*We were hoping to get sort of a champion, someone who would gather statistics and give feedback on a real timely basis when we do get patients who are admitted with stroke to see whether they received TPA, and if not, why not and answer questions like that.* (700SV3, 0)<br><br>*I guess [Stroke Director], I would see her as the closest to that. I guess just because she has been our point of contact and she does seem to have a real vested interest in making sure*	*I will fault my own leadership as being lacking. this was just an added responsibility. It wasn't like, you're going to get some support or some time to work on this stuff. I understand that resources are limited, but if you want a five-star meal you can't shop at Aldi, right? You've got to have some - you've got to spend some money and get some good ingredients.* (500SV1, -2)<br><br>*Part of the problem has been there has been a lot of bad blood between the administration and [CHIEF OF NEUROLOGY]. It’s personal sort of rift. So in a*

	rounds, but then also just to talk about any issues that arise. (200SV3, +2)	*the process runs smoothly. (700SV1, 0)*	*sense, I don't really like you, I'm not really going to support your stuff.* (1100SV2, -2)

DOMAIN: INTERVENTION

Complexity	Perceived difficulty of implementation, reflected by duration, scope, radicalness, disruptiveness, centrality, and intricacy and number of steps required to implement.
Design Quality & Packaging	Perceived excellence in how the intervention is bundled, presented, and assembled as noted from the staff evaluations.
Usability	Perceived degree of usefulness to the development and implementation of the local PREVENT program

DOMAIN: INNER SETTING – Local context

Construct	Short Description
Structural Characteristics	The social architecture, age, maturity, and size of an organization.
Networks & Communications	The nature and quality of webs of social networks and the nature and quality of formal and informal communications within an organization.
Culture	Norms, values, and basic assumptions of a given organization.
Goals & Feedback	The degree to which goals are clearly communicated, acted upon, and fed back to staff, and alignment of that feedback with goals.
Learning Climate	A climate in which: a) leaders express their own fallibility and need for team members' assistance and input; b) team members feel that they are essential, valued, and knowledgeable partners in the change process; c) individuals feel psychologically safe to try new methods; and d) there is sufficient time and space for reflective thinking and evaluation.
Leadership Engagement	Commitment, involvement, and accountability of leaders and managers with the implementation.
Available Resources	The level of resources dedicated for implementation and on-going operations, including money, training, education, physical space, and time.

DOMAIN: IMPLEMENTATION PROCESS

Construct	Short Description
Planning	The degree to which a scheme or method of behavior and tasks for implementing an intervention are developed in advance, and the quality of those schemes or methods.
Champions	Individuals who dedicate themselves to supporting, marketing, and ‘driving through’ an implementation, overcoming indifference or resistance that the intervention may provoke in an organization.
Reflecting & Evaluating	Quantitative and qualitative feedback about the progress and quality of implementation accompanied with regular personal and team debriefing about progress and experience.

Domain: INDIVIDUAL

Self-efficacy	One’s believe in their ability to perform a specific behavior

Categorical data captured by the analysis team will include individual-level data (e.g., job position, primary clinical area, etc.) for each of the interviews. We will use NVivo11 to integrate the tagged qualitative data, the scored construct data, the standardized survey data (e.g., OCAI measure), the categorical data, and the quantitative outcome data in a single, unified project file with multiple codebooks (qualitative, scored constructs, quantitative, categorical). We will use the NVivo11 “matrix query” feature to explore relationships between the qualitative, construct, quantitative and categorical data, and through this direct cross-referencing of qualitative and quantitative data iteratively analyze how specific combinations of intervention components, implementation strategies and features of local context influence project outcomes.

Aim 3 Hypothesis A: that sites that engage in ongoing reflecting and evaluating, goal setting, and planning will realize the greatest implementation success (based on the number of implementation activities completed and the level of team organization for providing and improving TIA care at 12 months).

As described above, the degree to which sites engage in reflecting and evaluating, goal setting, and planning will be assessed using an approach to qualitative coding of CFIR constructs that assigns valence and in this way each site will be assigned a designation that varies from -2 to +2 for the four constructs ((a) reflecting and evaluating, (b) goal setting, (c) planning, and (d) champions). The level of team organization is based on the GO score which varies from 1 (least complex level of organization) to 10 (most complex level of organization).

Example Table: Aim 3A

**Site**	**Final Construct Score** (possible range: -2 to +2)	**PRIMARY IMPLEMENTATION OUTCOMES**	

	Reflecting and Evaluating	Goal Setting	Planning	**Number of Improvement Initiatives Completed**	**Final GO Score**
A					
B					
C					
D					
E					
F					

Aim 3 Hypothesis B: that sites with greatest implementation success (based on the number of implementation activities completed and the level of team organization for providing and improving TIA care at 12 months) will achieve the highest Without-Fail rate. The measures of implementation success will be included in the GLMM model described above as cluster-level (aka facility-level) covariates. Specifically, the change in GO score from baseline to post-implementation as well as the number of implementation activities during the active implementation phase will be included in the model along with the covariate of the number of collaborative calls attended.

Example Table: Aim 3B

**Site**	**PRIMARY IMPLEMENTATION OUTCOMES**				**Final Without-Fail Rate**
	**Number of Implementation Activities Completed**	**Number of Collaborative Calls Attended**	**Final GO Score Provide**	**Final Go Score Improve**	
A					
B					
C					
D					
E					
F					

Aim 3 Secondary Implementation Analyses

Example Table: Aim 3C

**Strategies Used**	**Final Without Fail Rate**	
	**High**	**Low**
Reflecting (+/-)		
Goal Setting (+/-)		
Planning (+/-)		
Facilitation Dose		
Collaborative Calls (Dose)		
Use of Hub (Data Pages)		
Clinical Champion (scale rating)		
OCAI organizational profiles		

Identification of Effective Implementation Strategies

As described above, PREVENT employs three primary implementation strategies: (1) team activation via (a) audit and feedback, (b) reflecting and evaluating, (c) planning, and (d) goal setting; (2) external facilitation; and (3) building a community of practice. In addition, PREVENT allows for local adaptation of the intervention and also takes advantage of peer pressure.

The identification of successful implementation strategies will be based on a mixed methods assessment where the change in the Without-Fail rate will be used to define implementation success. As shown in Table 3C, we will compare facilities based upon a High Final Without Fail rate as defined as 50% or higher compared to those defined as low final rate (less than 50%) by the degree of engagement in implementation strategies and dose received as well as strength of clinical champion and organizational culture. Similarly, we will compare the rate of change [% of change] from baseline to end of active implementation.

External Facilitation

We will describe the dose, type, and temporal trends in external facilitation that is provided to each of the sites over the course of the one-year active implementation period. We will also ask participants to describe their experience with and their assessment of the external facilitation that was provided by the PREVENT program staff during the interviews at 6-months and 12-months post-implementation.

Community of Practice: Collaborative Call Assessment

Given that a key component of the implementation plan was the development of a virtual collaborative via monthly conference calls, we are particularly interested in the perceived value of the collaborative calls.

As part of the interviews at 6-months and 12-months post-implementation we will code the interviews to elicit the following emerging topics based upon the program element evaluation and motivation questions:

- Did the calls increase your utilization of the hub?
- Did the calls create a sense of profession community?
- Did the calls serve to maintain your interest and enthusiasm about TIA quality improvement?
- What was the single most important element of PREVENT participation for you working in your facility: kickoff, monthly process data on the hub, having access to the PREVENT community, the monthly calls, the library of resources and materials on the hub?

As part of the secondary implementation analysis we will examine whether participation in the calls (number of participants per site per call and presenting on the calls) relate to the Go Score. We will also examine whether hub usage (by date) increased just prior to or immediately following the calls. Similarly, we will examine whether email contact with the PREVENT team increased just prior to or immediately following a collaborative call. Finally, we will describe the "eureka moments" that occurred during or as a result of the calls.

Use of the HUB

Qualitative interview data from 6 months post-implementation and 1-year post-implementation will be used to understand how sites used the hub for data review and project planning. We will also identify whether and how the resources included in the hub library were used.

We will also examine data collected by the hub itself to identify the pages that are most commonly visited. Moreover, we will interview key users from each site with in depth usability questions.

4.4 Secondary Aim Methods (Sustainability)
The sustainability analysis will include: a comparison of the change in the Without-Rail rate from the baseline data period to the sustainability period (blue versus pink shading above). We will build multilevel models as described above for the Aim 2 analysis as described above.

We will explore whether sites with the greatest use of their own quality data (e.g., did they generate their own data, the frequency with which they reviewed the process of care data on the hub, whether they incorporated process of care data into routine work flow, did they conduct their own chart reviews) demonstrate the greatest program sustainability. Again, these cluster-level characteristics will be included in the GLMM model described above.

4.5 Risk Score
As part of the PREVENT program, participating sites are provided with their patient risk score which is a measure of the risk of 1-year mortality. We are interested in two analyses related to the risk score.

1. How did the sites use the patient risk score? This assessment will be based on qualitative interviews with sites at 6-months and 1-year post-implementation.

2. Risk score validation. For this analysis, all VAMCs will be included (not only PREVENT sites). Predicted risk scores, with their 95% confidence intervals, will be compared with observed 1-year mortality and its 95% confidence interval.

TABLE SHELLS

Table 1. Baseline Characteristics: at the Facility-Level

	**Baseline** (Control)	**Pre-Implementation**	**Post-Implementation** (Active Implementation)	**Sustainability**
Patient Characteristics				
Facility Characteristics				
90-Day Mortality Rate				
90-Day Recurrent Stroke Rate				
90-Day Recurrent TIA Rate				
90-Day Recurrent Stroke or TIA Rate				

Table 2. Unadjusted Effectiveness: at the Facility-Level
THIS IS THE PRIMARY ANALYSIS in terms of the data periods being compared.

	**Baseline (Control)**	**Post-Implementation** (Active Implementation)	**P-VALUE**
**Individual Processes of Care**			
Anticoagulation for Atrial Fibrillation			
Antithrombotics			
Brain Imaging			
Carotid Artery Imaging			
High/Moderate Potency Statin			
Hypertension Control			
Neurology Consultation			
**Mean Facility Consolidated Measure Rate**			
**Mean Facility Without-Fail Rate**			

Table 3. Hierarchical Model: Includes adjustment for patient and facility characteristics

The same approach described for the modelling the without-fail rate can be used to model the outcome rates.

Table 4. Temporal Trends in Quality of Care: Unadjusted

	**Baseline** (Control)	**Pre-Implementation**	**Post-Implementation** (Active Implementation)	**Sustainability**
**Individual Processes of Care**				
Anticoagulation for Atrial Fibrillation				
Antithrombotics				
Brain Imaging				
Carotid Artery Imaging				
High/Moderate Potency Statin				
Hypertension Control				
Neurology Consultation				
**Mean Facility Consolidated Measure Rate**				
**Mean Facility Without-Fail Rate**				

REFERENCES

1. Williams L. *Stroke Quality Enhancement Research Initiative (QUERI) Strategic Plan and Renewal Report.* 2013.
2. Johnston S, Gress D, Browner W, Sidney S. Short-term prognosis after emergency department diagnosis of TIA. *Jama.* 2000;284:2901-2906.
3. Johnston S. Short-term prognosis after a TIA: a simple score predicts risk. *Cleveland Clinic Journal of Medicine.* 2007;74(10):729-736.
4. Rothwell P, Johnston S. Transient ischemic attacks: stratifying risk. *Stroke; a journal of cerebral circulation.* 2006;37(2):320-322.
5. Kernan WN, Ovbiagele B, Black HR, et al. Guidelines for the prevention of stroke in patients with stroke and transient ischemic attack: a guideline for healthcare professionals from the American Heart Association/American Stroke Association. *Stroke; a journal of cerebral circulation.* 2014;45(7):2160-2236.
6. Bravata DM ML, Cheng E, Arling G, Miech EJ, Damush T, Sico J, Phipps M, Yu Z, Zillich A, Reeves M, Johanning J, Chatuvedi S, Baye F, Snow K, Barnd J, Slaven J, Austin C, Ferguson J, Livingston D, Maryfield B, Graham G, Rhude R, Williams LS. Quality of Care for Veterans with TIA and Minor Stroke. *Stroke; a journal of cerebral circulation.* 2015;46(Supp 1):ATMP73.
7. Lavallée PC, Meseguer E, Abboud H, et al. A transient ischaemic attack clinic with round-the-clock access (SOS-TIA): feasibility and effects. *Lancet Neurol.* 2007;6(11):953-960.
8. Luengo-Fernandez R, Gray AM, Rothwell PM. Effect of urgent treatment for transient ischaemic attack and minor stroke on disability and hospital costs (EXPRESS study): a prospective population-based sequential comparison. *Lancet Neurol.* 2009 8(3):235-243.
9. Rothwell PM, Giles MF, Chandratheva A, et al. Effect of urgent treatment of transient ischaemic attack and minor stroke on early recurrent stroke (EXPRESS study): a prospective population-based sequential comparison. *Lancet.* 2007 370(9596):1432-1442.
10. Lavallée P, Meseguer E, Abboud H, et al. A transient ischaemic attack clinic with round-the-clock access (SOS-TIA): feasibility and effects. *Lancet Neurol.* 2007;6(11):953-960.
11. Ranta A, Dovey S, Weatherall M, O'Dea D, Gommans J, Tilyard M. Cluster randomized controlled trial of TIA electronic decision support in primary care. *Neurology.* 2015;84:1545-1551.
12. Bosworth H, Olsen M, Grubber J, et al. Two self-management interventions to improve hypertension control: a randomized trial. *Annals of internal medicine.* 2009;151(10):687-695.
13. Bosworth H, Powers B, Olsen M, et al. Home blood pressure management and improved blood pressure control: results from a randomized controlled trial. *Archives of internal medicine.* 2011;171(13):1173-1180.
14. Kernan WN OB, Black HR, Bravata DM, Chimowitz MI, Ezekowitz MD, Fang MC, Fisher M, Furie KL, Heck DV, Johnston SC, Kasner SE, Kittner SJ, Mitchell PH, Rich MW, Richardson D, Schwamm LH, Wilson JA; American Heart Association Stroke Council, Council on Cardiovascular and Stroke Nursing, Council on Clinical Cardiology, and Council on Peripheral Vascular Disease. Guidelines for the prevention of stroke in patients with stroke and transient ischemic attack: a guideline for healthcare professionals from the American Heart Association/American Stroke Association. *Stroke.* 2014 45(7)::2160-2236.
15. Rothwell P, Giles M, Chandratheva A, et al. Effect of urgent treatment of transient ischaemic attack and minor stroke on early recurrent stroke (EXPRESS study): a prospective population-based sequential comparison. *Lancet (London, England).* 2007;370(9596):1432-1442.
16. Damush TM, Miech EJ, Sico JJ, et al. Barriers and facilitators to provide quality TIA care in the Veterans Healthcare Administration. *Neurology.* 2017;89(24):2422-2430.

17. Bravata DM, Myers LJ, Cheng E, et al. Development and Validation of Electronic Quality Measures to Assess Care for Patients With Transient Ischemic Attack and Minor Ischemic Stroke. *Circulation Cardiovascular quality and outcomes.* 2017;10(9):pii: e003157.
18. Bravata D, Myers L, Arling G, et al. The Quality of Care for Veterans with Transient Ischemic Attack and Minor Stroke. *JAMA Neurology.* 2017.
19. Proctor E, Silmere H, Raghavan R, et al. Outcomes for implementation research: conceptual distinctions, measurement challenges, and research agenda. *Administration and policy in mental health.* 2011;38(2):65-76.
20. Brown CA, Lilford RJ. The stepped wedge trial design: a systematic review. *BMC medical research methodology.* 2006;6:54.
21. Mdege ND, Man MS, Taylor Nee Brown CA, Torgerson DJ. Systematic review of stepped wedge cluster randomized trials shows that design is particularly used to evaluate interventions during routine implementation. *J Clin Epidemiol.* 2011;64(9):936-948.
22. Miech E. *The GO Score: A New Context-Sensitive Instrument to Measure Group Organization Level for Providing and Improving Care.* Washington DC.2015.
23. Hamilton A, Brunner J, Cain C, et al. Engaging multilevel stakeholders in an implementation trial of evidence-based quality improvement in VA women's health primary care. *TBM.* 2017; 7(3):478-485.
24. Miech ED, TM. The Consolidated Framework for Implementation Research: Applying the CFIR Constructs Directly to Qualitative Data. Bi-Annual Meeting: Society for Implementation Research Collaboration; 2015; Seattle, Washington.
25. Miech ED, TM. Applying the CFIR Constructs Directly to Qualitative Data: The Power of Implementation Science In Action. HSR&D/QUERI National Conference; 2015; Philadelphia, PA.
26. Damush T, Damschroder L. CFIR Implementation Framework with Application to the VISN11 Stroke Collaborative. *VA Cyber-Seminar*2010.
