# Supplementary material for: Assessment of the Protocol-Guided Rapid Evaluation of Veterans Experiencing New Transient Neurological Symptoms (PREVENT) Program for Improving Quality of Care for Transient Ischemic Attack: A Nonrandomized Cluster Trial
Source: JAMA Netw Open. 2020 Sep 8;3(9):e2015920. doi: 10.1001/jamanetworkopen.2020.15920 (PMC7489850; doi:10.1001/jamanetworkopen.2020.15920)
Supplement: Supplement 2. — eTable 1. Definitions of Seven Processes of Care Included in the Without-Fail Measure eTable 2. Diagnosis Codes eTable 3. Risk Adjustment Variables for Each Process Measure or Outcome eTable 4. Facility Staffing for Matched Control and PREVENT Sites eTable 5. Without Fail Rates by Facility and Study Period eTable 6. Patient Outcomes at PREVENT Sites and Matched Control Sites eFigure 1. Time to Recurrent Events: Disease-Free Survival eFigure 2. Illustration of the Without-Fail Rate for Two Hypothetical Facilities [file jamanetwopen-e2015920-s002.pdf]

Supplementary Online Content

Bravata DM, Myers LJ, Perkins AJ, et al. Assessment of the Protocol-Guided Rapid Evaluation of Veterans Experiencing New Transient Neurological Symptoms (PREVENT) program for improving quality of care for transient ischemic attack: a nonrandomized cluster trial. *JAMA Netw Open*. 2020;3(9):e2015920. doi:10.1001/jamanetworkopen.2020.15920

eTable 1. Definitions of Seven Processes of Care Included in the Without-Fail Measure

eTable 2. Diagnosis Codes

eTable 3. Risk Adjustment Variables for Each Process Measure or Outcome

eTable 4. Facility Staffing for Matched Control and PREVENT Sites

eTable 5. Without Fail Rates by Facility and Study Period

eTable 6. Patient Outcomes at PREVENT Sites and Matched Control Sites

eFigure 1. Time to Recurrent Events: Disease-Free Survival

eFigure 2. Illustration of the Without-Fail Rate for Two Hypothetical Facilities

This supplementary material has been provided by the authors to give readers additional information about their work.

© 2020 Bravata DM et al. *JAMA Network Open*.

eTable 1. Definitions of Seven Processes of Care Included in the Without-Fail Measure

Label	Numerator	Denominator	Exclusions
Anticoagulation for Atrial Fibrillation/Flutter	Patients prescribed anticoagulation therapy within 7 days after discharge from Emergency Department (ED) or hospital.<br><br>Outpatient medications prior to presentation may be used if anticoagulants are not provided or prescribed within 7 days of discharge. If anticoagulants are not received within 7 days of discharge, then look in the 120 days prior to presentation and confirm the day supply extends into the 7 days after discharge. A 30-day buffer is added to outpatient day-supply to account for possible stockpiling.	TIA patients with atrial fibrillation/paroxysmal atrial fibrillation atrial fibrillation/ in prior 5-year or diagnosed prior to discharge	• Patients without a history of atrial fibrillation/flutter<br>• Transferred to a Non-VA acute care facility<br>• Patients who died during hospital stay or ED visit<br>• Patients who left against medical advice (AMA)<br>• Patients discharged to hospice<br>• Patients with an allergy to any anticoagulant<br>• Patients who receive thrombolysis (tPA) within 2 days of discharge<br>• Patients on dialysis 6 months pre-event, during the hospital stay or within 7days after the event<br>• Patients with history of atrial ablation in 5 years prior to event<br>• History of liver disease with most recent international normalized ration (INR) > 1.7<br>• History of hepatocellular carcinoma in prior 1-Year<br>• Patients with ≥1 admission for major bleeding event in 1-year prior to index date<br>• History of liver disease with alanine aminotransferase (ALT) > 120 U/L in prior 1-Year<br>• Palliative care or Hospice in 1-Year prior to presentation<br>• Patients with history of intracerebral hemorrhage (ICH) in 1-year prior to index date<br>• Patients with history of cerebral amyloid angiopathy (CAA) in 1-year prior to index date
Antithrombotics	Patients who receive antithrombotic therapy by the end of day two after presentation (where the day of presentation is considered day-1, and the following day is considered day-2)<br><br>Outpatient medications prior to presentation may be used if antithrombotics are not provided or prescribed by day two. If antithrombotics are not received by day two, then look in the 120 days prior to presentation and confirm the day supply extends into day two. A 30-day buffer is added to outpatient day-supply to account for possible stockpiling.	TIA patient cohort	• Died on day of or day following presentation<br>• Discharged to hospice by day two<br>• Transferred to another Non-VA acute care facility by day 2<br>• Patients who left against medical advice by day 2<br>• Patients with an allergy to antithrombotic medication, noted prior to end of day 2<br>• Patients who receive tPA by day 2<br>• Palliative care or Hospice in 1-Year prior to presentation<br>• Patients with ≥1 admission for major bleeding event in 1-year prior to index date<br>• Patients with history of intracerebral hemorrhage (ICH) in 1-year prior to index date<br>• Patient with history of cerebral amyloid angiopathy (CAA) in 1-year prior to index date

© 2020 Bravata DM et al. *JAMA Network Open*.

eTable 1. (continued)

Brain Imaging	Patients receiving brain imaging (Brain computed tomography [CT] or magnetic resonance imaging [MRI]) within 2 days of index event or 1 day prior to index event	TIA patient cohort	• Died within two days of index event<br>• Discharged to hospice within 2 days of index event<br>• Left AMA within 2 days of index event<br>• Transferred to a Non-VA acute care facility within 2 days of index event<br>• Admitted from a Non-VA acute care facility
Carotid Artery Imaging	Patients receiving a carotid imaging procedure within 2 days of index event or 6 months prior to index event or who have an order for carotid image within 2days after index event	TIA patient cohort	• Died within 2 days of index event<br>• Transferred to another Non-VA acute care facility within 2 days of index event<br>• Discharged to hospice<br>• Palliative care or Hospice in 1-Year prior to index event<br>• Patients who left against medical advice within 2 days after index event
High or Moderate Potency Statin at Discharge	• Patients who receive outpatient statin therapy within 7 days after discharge (with >1 day of supply) defined as follows:<br>○ Moderate or High Potency: >75 years, 2 LDL values <40 mg/dL over a minimum of at least 8-week time period prior to index, Asian Race, history of hemorrhagic stroke or myopathy or solid organ transplant in prior year, HIV positive<br>○ High Potency Statin ≤75 years of age without any of the other reasons for moderate potency listed above<br>Use outpatient medications in 7 days following discharge to identify the statin; If no statins are prescribed within 7-days of discharge, then look in the 120 days before presentation and confirm the day supply extends into the 7 day post discharge period. A 30-day buffer is added to outpatient day-supply to account for possible stockpiling.	TIA patient cohort	• Transferred to another Non-VA acute care facility<br>• Patients who died during the hospital stay or during ED visit<br>• Patients discharged to hospice<br>• Patients who left against medical advice (AMA)<br>• Patients with allergy to statin therapy<br>• Patients on anti-rejection medication in 1-year prior to index<br>• Patients on hepatitis C medication in 1-year prior to index (interferon, sofosbuvir, ribavirin, simeprevir, ledipasvir)<br>• Patients on dialysis 6 months pre-event or prior to discharge or within 7days after discharge<br>• Patients with severe congestive heart failure (CHF, defined as≥2 inpatient admission with primary diagnosis of CHF in 1-year prior to index event)<br>• Pregnant Woman<br>• Patients with liver disease and ALT > 120 U/L in 1-year prior to index<br>• Palliative care or Hospice in 1-Year prior to presentation<br>• Patients with history of intracerebral hemorrhage (ICH) in 1-year prior to index date<br>• Patient with history of cerebral amyloid angiopathy (CAA) in 1-year prior to index date
Hypertension Control	Patients whose calculated mean blood pressure from the values in the 90 days after discharge is systolic blood pressure <140 mm Hg **and** diastolic blood pressure <90 mm Hg. Only outpatient BP from clinics designated as being responsible for hypertension are included (e.g., primary care, neurology, cardiology).	TIA patient cohort	• Died during index TIA hospitalization or within 90 days of discharge<br>• Discharged to hospice<br>• Transferred to another Non-VA acute care facility<br>• Patients on dialysis 6 months pre-event, during the hospital stay or within 90 days after the event<br>• On ≥4 antihypertensive medications in 90days prior to index event<br>• Left AMA<br>• Pregnant Woman<br>• Palliative Care or Hospice in 1-Year prior to presentation
Neurology Consult	Patient who receive a Neurology consult on day of presentation or day following presentation using orders, consults, bed section, clinic visit, note titles, or neurologist provider codes.	TIA patient cohort	• Transferred to another Non-VA acute care facility within 1 days of index event<br>• Left AMA within 1-day<br>• Palliative Care or Hospice in 1-Year prior to presentation

© 2020 Bravata DM et al. *JAMA Network Open*.

eTable 2. Diagnosis Codes

CHF	MI or ACS	Ventricular Arrhythmia	Stroke	TIA
398.91<br>402.01<br>402.11<br>402.91<br>404.01<br>404.03<br>404.11<br>404.13<br>404.91<br>404.93<br>425.4<br>425.5<br>425.6<br>425.7<br>425.8<br>425.9<br>I09.9<br>I11.0<br>I13.0<br>I13.2<br>I25.5<br>I42.0<br>I42.5<br>I42.5<br>I42.6<br>I42.7<br>I42.8<br>I42.9<br>I43<br>I50<br>P29.0	410<br>411<br>412<br>I20<br>I21<br>I22<br>I25.2	427 (not 427.31 or 427.32)<br>I47<br>I49<br>I46<br>R00	436<br>433.01<br>433.11<br>433.21<br>433.31<br>433.81<br>433.91<br>434.00<br>434.01<br>434.11<br>434.21<br>434.31<br>348.1<br>434.91<br>I63<br>I66<br>I67.89<br>I97.81<br>I97.82	435 (not 435.2)<br>G45.0<br>G45.1<br>G45.8<br>G45.9<br>I67.848
Given that these codes were used both to identify past medical history based on events before the index TIA as well as prospectively to identify recurrent vascular events, they include both ICD-9 and ICD-10 codes.				

© 2020 Bravata DM et al. *JAMA Network Open*.

eTable 3. Risk Adjustment Variables for Each Process Measure or Outcome

Outcome	Risk Adjustment Variables
Anticoagulation for Atrial Fibrillation	Age, History of Atrial Fibrillation, History Intracranial Hemorrhage, HASBLED score, Warfarin
Antithrombotics	History of TIA (outpatient encounter), History of Atrial Fibrillation, Hemiplegia, Aspirin, Warfarin, Clopidogrel
Brain Imaging	Apache, History of TIA (outpatient encounter)
Carotid Artery Imaging	Age, History of TIA (outpatient encounter), History of Cerebral Amyloid Angiopathy, History of Chronic Kidney Disease, Hemiplegia, Amaurosis Fugax, Aspirin, Statin, Clopidogrel
High/Moderate Potency Statin	History of TIA (outpatient encounter), History of Chronic Kidney Disease, History of Cirrhosis, History of Valvular Disease, Aspirin, Statin, Clopidogrel
Hypertension Control	Age, Systolic Blood Pressure, Apache, History of Hypertension, History of Hyperlipidemia, History of Arrhythmia, Antihypertensive Medications
Neurology Consultation	Age, History of TIA (outpatient encounter), Hemiplegia, Amaurosis Fugax, Statin, Clopidogrel
Consolidated Rate	Age, History of TIA (outpatient encounter), Hemiplegia, History of Dialysis, Amaurosis Fugax, HASBLED Score, Palliative/Hospice Care, Aspirin, Warfarin, Statin, Clopidogrel
Without Fail Rate	Age, History of TIA (outpatient encounter), Hemiplegia, History of Dialysis, Amaurosis Fugax, HASBLED Score, Palliative/Hospice Care, Aspirin, Warfarin, Statin, Clopidogrel
Death within 90 days of discharge	Age, Gender, Race, Charlson Comorbidity Index, Palliative/Hospice Care, History of Atrial Fibrillation, Hemiplegia, Syncope, Concomitant CHF, Systolic Blood Pressure
Death or stroke within 90 days of discharge	Age, Gender, Race, APACHE, Charlson Comorbidity Index, Current Smoker, Palliative/Hospice Care, History of TIA (outpatient encounter), History of Stroke, History of Depression, History of Intracranial Hemorrhage, Hemiplegia, Systolic Blood Pressure
Recurrent vascular event within 90 days of discharge	Age, Gender, Race, APACHE, Charlson Comorbidity Index, Current Smoker, Palliative/Hospice Care, History of TIA (outpatient encounter), History of Stroke, History of Depression, History of Intracranial Hemorrhage, Hemiplegia, Systolic Blood Pressure

© 2020 Bravata DM et al. *JAMA Network Open*.

eTable 4. Facility Staffing for Matched Control and PREVENT Sites

Facility Staffing	Matched Control Sites	PREVENT Sites	P-value
**Neurology FTE***			
Mean (SD)†	3.4 (1.9)	4.7 (1.4)	0.117
Median (IQR)§	3.2 (1.9–5.0)	4.5 (3.6–5.8)	0.089
**ED FTE***			
Mean (SD) †	5.3 (4.2)	7.1 (1.0)	0.321
Median (IQR)§	4.4 (2.5–7.7)	6.9 (6.4–7.3)	0.153

*FTE refers to full-time equivalent

†SD refers to standard deviation.

§IQR refers to the interquartile range.

© 2020 Bravata DM et al. *JAMA Network Open*.

eTable 5. Without Fail Rates by Facility and Study Period

Facility	PREVENT Sites	
	**Baseline Period**	**Implementation Period**
A	16.3% (7/43)	34.5% (10/29)
B	33.3% (6/18)	44.4% (12/27)
C	38.5% (5/13)	60.0% (6/10)
D	38.7% (12/31)	52.2% (35/67)
E	50.0% (12/24)	78.3% (18/23)
F	55.2% (16/29)	70.0% (14/20)

© 2020 Bravata DM et al. *JAMA Network Open*.

eTable 6. Patient Outcomes at PREVENT Sites and Matched Control Sites†

90-Day Outcome	Control Sites			PREVENT Sites			Unadjusted			Adjusted		
	Baseline	Implementation	Change	Baseline	Implementation	Change	Control	PREVENT	Interaction	Control	PREVENT	Interaction
	Pass/Eligible (%)			Pass/Eligible (%)			OR (95% CI)		P-Value	OR (95% CI)		P-Value
All-cause mortality	22/973 (2.3)	16/968 (1.7)	-0.6%	4/162 (2.5)	3/189 (1.6)	-0.9%	0.73 (0.38, 1.40)	0.67 (0.15, 3.09)	0.922	0.72 (0.34, 1.49)	0.88 (0.16, 4.70)	0.825
Stroke	33/952 (3.5)	29/954 (3.0)	-0.5%	3/158 (1.9)	2/186 (1.1)	-0.8%	0.89 (0.53, 1.47)	0.58 (0.10, 3.53)	0.656	0.89 (0.53, 1.49)	0.57 (0.09, 3.51)	0.646
Stroke or death	54/973 (5.5)	43/968 (4.4)	-1.1%	7/162 (4.3)	5/189 (2.6)	-1.7%	0.80 (0.53, 1.21)	0.61 (018, 1.99)	0.673	0.79 (0.51, 1.21)	0.61 (0.18, 2.07)	0.700
Recurrent event*	115/973 (11.8)	96/968 (9.9)	-1.9%	16/162 (9.9)	16/189 (8.5)	-1.4%	0.84 (0.62, 1.12)	0.86 (0.41, 1.80)	0.935	0.83 (0.61, 1.12)	0.94 (0.44, 2.03)	0.753

*The recurrent event endpoint included: congestive heart failure, myocardial infarction/acute coronary syndrome, ischemic stroke, TIA, ventricular arrhythmia, or death from any cause

†The PREVENT project was not powered to detect differences in patient outcome rates.

© 2020 Bravata DM et al. *JAMA Network Open*.

eFigure 1. Time to Recurrent Events: Disease-Free Survival

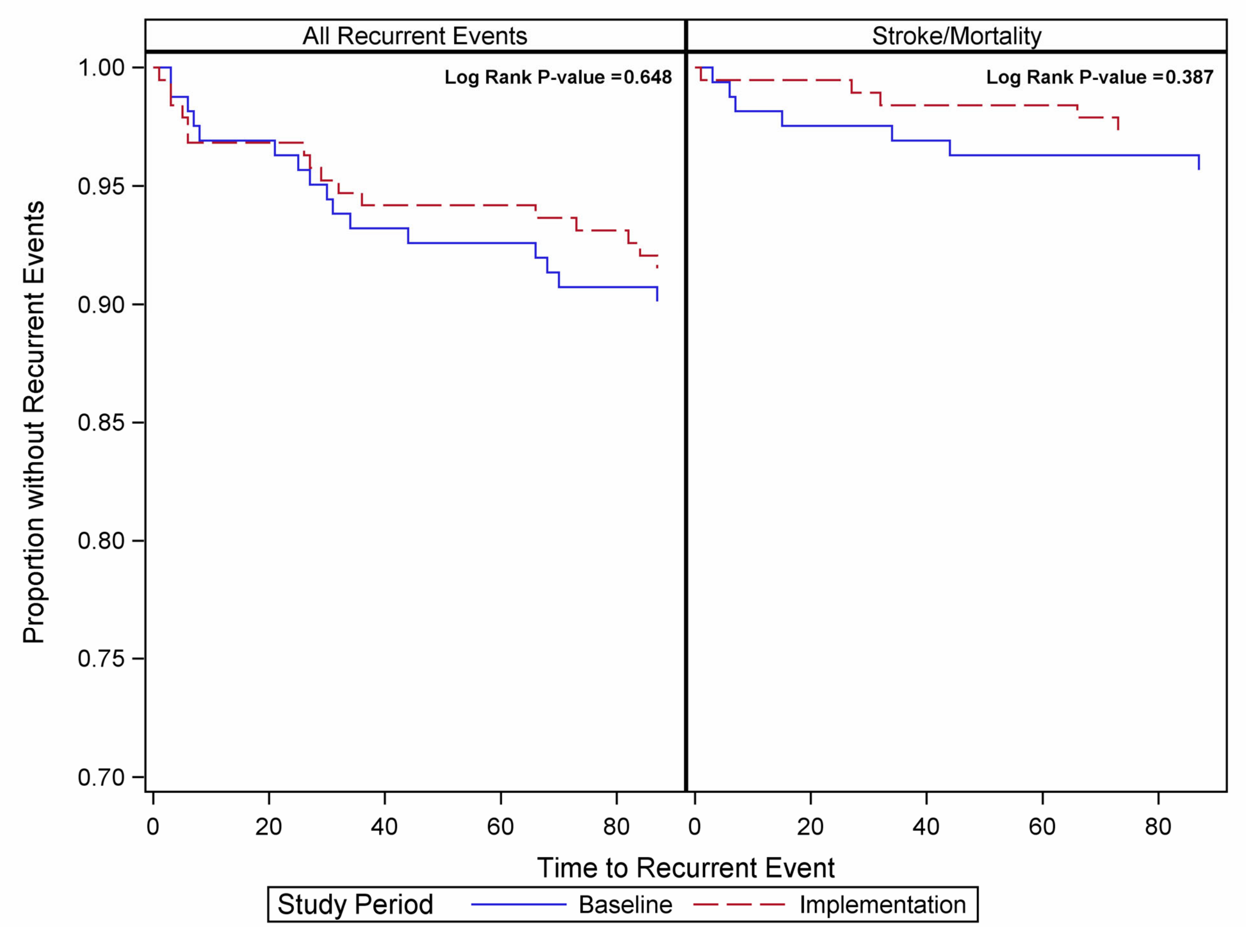

FIGURE 1 LEGEND

The figure displays along the horizontal axis the time in days from the index transient ischemic attack (TIA) to any recurrent event (left panel) and to ischemic stroke or death (in the right panel); the vertical axis displays the proportion of patients without any outcome event (disease-free survival). Data are provided for patients in PREVENT sites during the baseline period (blue solid line) and the intervention period (red dashed line).

© 2020 Bravata DM et al. *JAMA Network Open*.

eFigure 2. Illustration of the Without-Fail Rate for Two Hypothetical Facilities

Patients within hypothetical facility A	Processes of Care							Number of Processes Passed/ Number Eligible	Without-Fail Rate*	
	1	2	3	4	5	6	7		Patient	Facility
1st Patient	✓	✓	✓	✓	✓	✓	✓	**7/7**	100%	**100%** (1/1)
2nd Patient	✓	✓	✓	✓	✓	✓	Not eligible	**6/6**	100%	**100%** (2/2)
3rd Patient	✗	✓	✓	✓	Not eligible	✓	✓	**5/6**	0%	**66.7%** (2/3)
4th Patient	✓	✓	✓	Not eligible	✓	✗	✗	**4/6**	0%	**50%** (2/4)
5th Patient	✓	✓	✓	✓	✗	Not eligible	Not eligible	**4/5**	0%	**40%** (2/5)

Patients within hypothetical facility B	Processes of Care							Number of Processes Passed/ Number Eligible	Without-Fail Rate*	
	1	2	3	4	5	6	7		Patient	Facility
1st Patient	✓	✓	✓	✓	✓	✓	✓	**7/7**	100%	**100%** (1/1)
2nd Patient	✓	✓	✓	✓	✓	✓	Not eligible	**6/6**	100%	**100%** (2/2)
3rd Patient	✗	✓	✓	✓	Not eligible	✓	✓	**5/6**	0%	**66.7%** (2/3)
4th Patient	✗	✓	✓	Not eligible	✓	✓	✓	**5/6**	0%	**50%** (2/4)
5th Patient	✗	✓	✓	✓	✓	Not eligible	Not eligible	**4/5**	0%	**40%** (2/5)

FIGURE 2 LEGEND

*The without-fail rate is the proportion of patients who received all of the care for which they were eligible among seven guideline-concordant processes of care.

✓ refers to passing a process of care.

✗ refers to failing a process of care.

© 2020 Bravata DM et al. *JAMA Network Open*.
