# Supplementary material for: Assessment of the Protocol-Guided Rapid Evaluation of Veterans Experiencing New Transient Neurological Symptoms (PREVENT) Program for Improving Quality of Care for Transient Ischemic Attack: A Nonrandomized Cluster Trial
Source: JAMA Netw Open. 2020 Sep 8;3(9):e2015920. doi: 10.1001/jamanetworkopen.2020.15920 (PMC7489850; doi:10.1001/jamanetworkopen.2020.15920)
Supplement: Supplement 3. — Data Sharing Statement [file jamanetwopen-e2015920-s003.pdf]

Data Sharing Statement

Bravata. Assessment of the Protocol-Guided Rapid Evaluation of Veterans Experiencing New Transient Neurological Symptoms (PREVENT) Program for Improving Quality of Care for Transient Ischemic Attack. *JAMA Netw Open*. Published September 8, 2020. 10.1001/jamanetworkopen.2020.15920

Data available: No

Explanation for why data not available: These data must remain on Department of Veterans Affairs servers. Investigators interested in using these data for analyses should email the corresponding author.
